# Enrichment of centromeric DNA from human cells

**DOI:** 10.1371/journal.pgen.1010306

**Published:** 2022-07-19

**Authors:** Riccardo Gamba, Giulia Mazzucco, Therese Wilhelm, Leonid Velikovsky, Catalina Salinas-Luypaert, Florian Chardon, Julien Picotto, Mylène Bohec, Sylvain Baulande, Ylli Doksani, Daniele Fachinetti

**Affiliations:** 1 Institut Curie, PSL Research University, CNRS, UMR 144, Paris, France; 2 IFOM, the FIRC Institute of Molecular Oncology, Milan, Italy; 3 Institut Curie, Genomics of Excellence (ICGex) Platform, PSL Research University, Paris, France; Peking University, CHINA

## Abstract

Centromeres are key elements for chromosome segregation. Canonical centromeres are built over long-stretches of tandem repetitive arrays. Despite being quite abundant compared to other loci, centromere sequences overall still represent only 2 to 5% of the human genome, therefore studying their genetic and epigenetic features is a major challenge. Furthermore, sequencing of centromeric regions requires high coverage to fully analyze length and sequence variations, and this can be extremely costly. To bypass these issues, we have developed a technique, named CenRICH, to enrich for centromeric DNA from human cells based on selective restriction digestion and size fractionation. Combining restriction enzymes cutting at high frequency throughout the genome, except within most human centromeres, with size-selection of fragments >20 kb, resulted in over 25-fold enrichment in centromeric DNA. High-throughput sequencing revealed that up to 60% of the DNA in the enriched samples is made of centromeric repeats. We show that this method can be used in combination with long-read sequencing to investigate the DNA methylation status of certain centromeres and, with a specific enzyme combination, also of their surrounding regions (mainly HSATII). Finally, we show that CenRICH facilitates single-molecule analysis of replicating centromeric fibers by DNA combing. This approach has great potential for making sequencing of centromeric DNA more affordable and efficient and for single DNA molecule studies.

## Author summary

Centromeres are the portions of the chromosomes required for the correct partitioning of genetic material into the daughter cells. In humans, centromeric DNA is made of highly repetitive DNA sequences that hindered its precise molecular characterization until very recently with the development of pivotal technological advances. However, these approaches require the analysis of the whole human genome, while centromeres only represent less than 5%. For this reason, detailed characterization of human centromeres is still very expensive in terms of cost, timing and data analysis. We propose a method called CenRICH that allows to enrich and purify for human centromeric DNA. We prove that this method provides several advantages: 1) it drastically reduces the cost of centromere sequencing; 2) it can be used to study the epigenetic status of centromeres with high level of resolution; 3) it is suitable for single molecule visualization with advanced microscopy techniques. Therefore, CenRICH is a powerful tool to facilitate many future studies in the ever-expanding field of centromere biology, with potential application in study of genetic disease.

## Introduction

Centromeres are the chromosomal sites for assembly of kinetochores, the fundamental complex necessary for proper chromosome segregation in both meiosis and mitosis [1, 2]. In humans they are composed of highly repetitive arrays of alpha satellite DNA (α-sat) that stretches over megabase-long regions [3]. α-sat DNA is organized in head-to-tail tandem repeats of single AT-rich 171 bp monomers that can form highly homogeneous Higher Order Repeat (HOR) units of different length and composition among different chromosomes. These HORs are typically flanked by monomeric divergent alpha satellite repeats, and different HOR arrays on the same centromere can be separated by other repeat families [4–7].

Centromeric DNA and its DNA binding protein CENP-B have been recently implicated in centromere stability or function [1, 8–12]. Yet, the repetitive nature of these loci has hindered their detailed molecular characterization. The use of novel, long-read sequencing approaches and the development of new computational methods has recently allowed a breakthrough in the dissection of the sequence of these long repetitive regions. This is exemplified by the recent release of a whole uninterrupted telomere to telomere (T2T) sequence of a human genome (from a hydatidiform mole derived cell line, CHM13-hTERT, hereafter called CHM13) [4–7]. These advances in DNA sequencing and mapping open a new era in the genomic study of centromeres. Nevertheless, probing centromeric DNA still poses some difficulties, especially considering that centromeric repeats can vary across individuals and between homologous chromosomes.

A major limitation in the study of centromeric DNA is that there are no widely established and efficient methods to select centromeric regions and isolate them from the rest of the genome. Therefore, investigation of the centromeric sequence requires whole genome sequencing (WGS), a very inefficient and costly approach as only 2–5% of the human genome is composed by centromeric DNA [7, 13]. Furthermore, the study of centromere replication and structure with single-molecule imaging methods is limited by the usage of fluorescent probes to identify centromeric DNA. Labeling is not always feasible (e.g. it is not compatible with electron microscopy) and when it is (e.g. DNA combing), it requires long acquisition and analysis time since only 2–5% of the molecules are labelled as centromeric.

Use of immuno-precipitation methods relying on the presence of centromeric proteins can only isolate a sub-portion of the whole centromeric α-sat arrays. According to recent estimates, CENP-A, the histone H3 variant enriched at centromeric regions [14], spans a region of approximately 0.2 to 0.5 Mb per centromere, totaling to ~7.8Mb, less than 10% of the α-sat content in the genome [7]. Also, immuno-precipitation methods do not provide long, uninterrupted DNA fragments that are necessary to unravel the centromere sequence and structure.

Another approach to enrich for a target sequence is based on restriction enzymes and relies on the digestion of the rest of the genome while maintaining the regions of interest largely intact. This rationale is applied for the purification of telomeric repeats, which lack canonical restriction sites [15–17]. More recently, a two-step procedure has been developed for the study of telomere structure by electron microscopy (EM) [18, 19]. While a similar restriction-based approach was developed in the pre-genomic era to isolate mouse (peri)centromeres [20], an analogous widely established technique for the study of human centromeres is currently missing.

**Fig 1:**
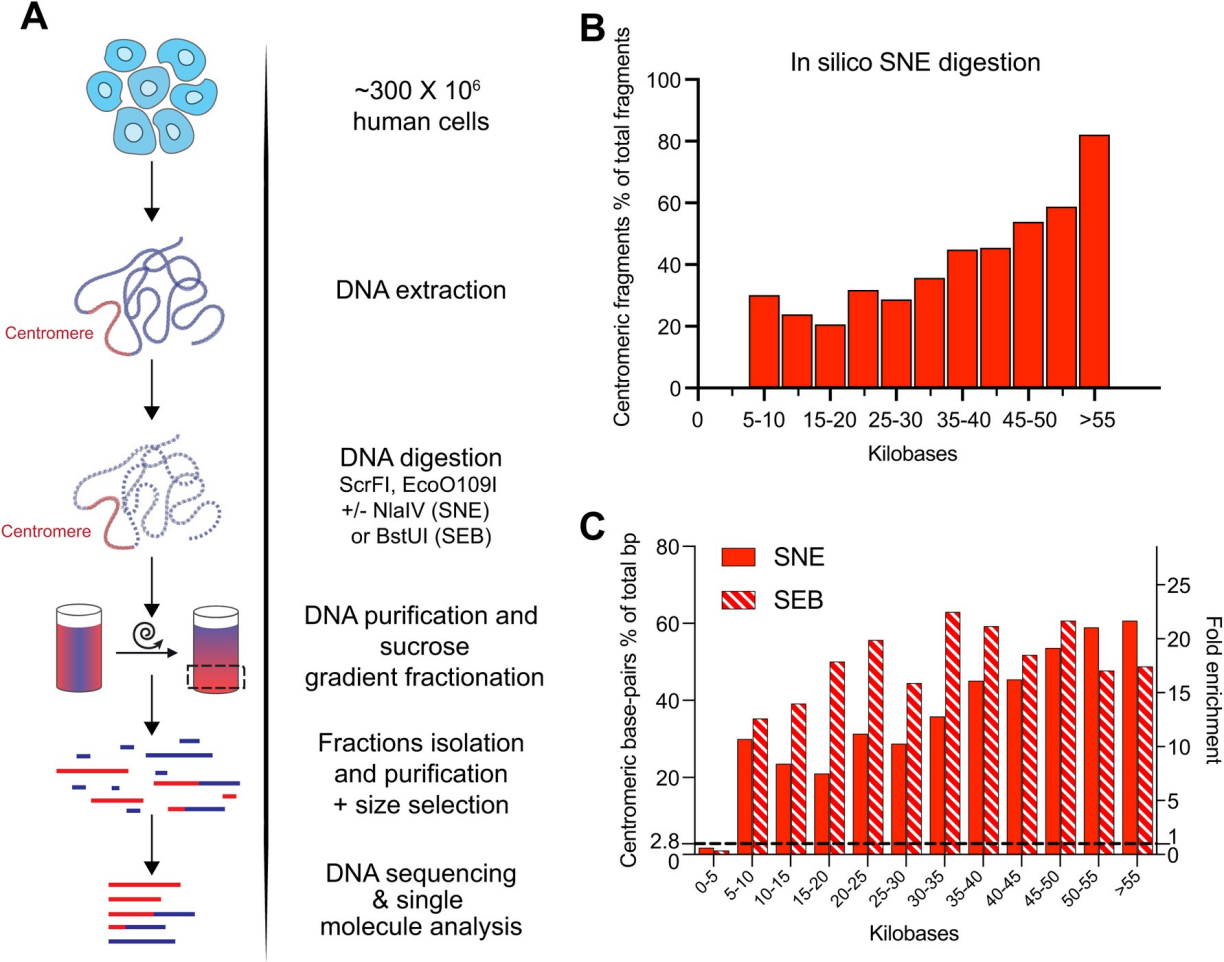
A restriction enzyme-based method to enrich and purify centromeric DNA from human cells. **A**. Schematic representation of the experimental design. **B**. Predicted distribution of the percentage of centromeric fragments in the indicated size bins after *in silico* digestion of the reference T2T-CHM13v1.0 genome with the SNE enzyme combination. Y-axis represents the percentage of centromeric fragments over total fragments in each length range. **C**. Distribution of centromeric base-pairs according to predicted fragment length after *in silico* digestion of the reference T2T-CHM13v1.0 genome with the SNE or SEB enzyme combinations. The y-axis on the left represents the percentage of centromeric base-pairs over total base-pairs in each length range. The dotted line at 2.8% represents the percentage of centromeric base-pairs in the reference genome, corresponding to the expected fraction of centromeric DNA in a theoretical non-enriched sample. The y-axis on the right reports the fold enrichment in centromeric base-pairs over the non-enriched sample (~2.8% of centromeric base-pairs in the reference genome).

In this manuscript, we present the development of a restriction digestion-based method to enrich for centromeric repeats, and with certain enzyme combinations also for pericentromeric satellites, that allows isolation of high molecular weight (HMW), long fragments of centromeric DNA suitable for long-read sequencing (**Fig 1A**). Our method, named CenRICH, drastically increases the efficiency of centromeric DNA sequencing compared to whole genome sequencing, facilitating its downstream genetic and epigenetic analysis. Furthermore, we demonstrate that this method allows direct visualization of long centromeric fragments in fluorescence microscopy, with possible applications for single-molecule analysis of centromeric DNA.

## Description of the method

The method relies on the extraction of large quantities of genomic DNA, digestion with three restriction enzymes followed by size fractionation of the fragments with sucrose gradient centrifugation. The high molecular weight fractions are then recovered and used for downstream applications.

### DNA extraction and digestion

To obtain a centromere-enriched sample to be analyzed with multiple techniques, 2.5 to 3 mg of genomic DNA was extracted from 300–400 million cells, as previously described [18, 19]. When less enriched DNA is needed, (e.g. for use only in DNA combing) we have scaled down our method starting from about 100-150M cells, extracting about 700–800 μg of genomic DNA and obtaining about 20 μg of enriched DNA. Briefly:

1. Cells were trypsinized, washed twice in PBS 1X and resuspended in TNE buffer (10 mM Tris-HCl pH 7.4, 1mM EDTA pH8, 100 mM NaCl).
2. Cells were lysed by adding one volume of TNES buffer (TNE + 1% SDS) supplemented with RNaseA (Invitrogen cat #12091021) at final concentration of 100 μg/mL and incubated at 37° for 30 minutes.
3. Proteinase K treatment (Invitrogen cat #25530049) was performed overnight at 37° at a final concentration of 100 μg/ml.
4. DNA was extracted with one volume of Phenol:Chloroform:Isoamylalcol (25:24:1) (Sigma Aldrich cat#77617). After centrifugation at 3500 g for 5 minutes, one volume of chloroform was added to the aqueous phase.
5. After centrifugation at 3500 g for 5 minutes, the DNA in the aqueous phase was precipitated with 0.1 volume of sodium acetate 3M pH 5.2 and one volume of Isopropanol.
6. After washing with 70% ethanol, DNA was gently resuspended in 1 ml of Tris-HCl 10 mM pH 8.0.
7. 2.5 mg of DNA were resuspended in 20 mL of 1X CutSmart Buffer (NEB cat#B7204S) and incubated at RT for one hour on a rotating wheel.
8. Digestion was carried out over night at 37° using 400 units each of ScrFI and EcoO109I and with 400 units of NlaIV or BstUI (New England Biolabs). When applicable, 1 μM of T-EN enzyme (telomere digesting) was added to the digestion mix [21].
9. Digestion products were purified with one step of phenol:chloroform:isoamylalcol (25:24:1) purification and precipitated with isopropanol and sodium acetate, as above. DNA was resuspended in 4.5 mL of TE 1X.

### Sucrose gradient fractionation

1. Sucrose gradients were prepared with 8 ml each of 40%, 30% and 20% sucrose solutions in TNE buffer, carefully deposited sequentially on top of each other in Thickwall, Ultra-Clear tubes (Beckman Coulter cat #344058) compatible with SW32Ti rotor.
2. The digested DNA sample was split in 4 aliquots, each in a volume of 1.5 ml, and incubated at 50° for 5 minutes prior to loading each aliquot on a separate sucrose gradient.
3. The gradients were centrifuged at 4° in a SW32Ti rotor at 30100 rpm for 16 hours.
4. The fractions were collected as follows: the top 5.5 ml were collected as fraction 1 (F1) while the remaining F2 to F6 consisted of 4 ml each.
5. Fractions were concentrated using Amicon Ultra 15 ml centrifugal filters (MWCO = 30 kDa, Merck, cat# UFC903024) performing 5–6 washes of the filter with Tris-HCl 10 mM pH 8.0. The sample (0.5–1 ml) was transferred to Amicon Ultra 0.5 ml Centrifugal Filters (MWCO = 30 kDa, Merck, cat# UFC503096) and further concentrated to a final volume of 200 μl.

### Cell lines

All cells were maintained at 37°C in a 5% CO_2_ atmosphere. Immortalized hTERT RPE-1 cells were cultured using DMEM:F12 medium containing 10% Fetal Bovine Serum (BioSera), 0.123% sodium bicarbonate, and 2 mM L-glutamine. DLD-1 and HCT116 cells [22] were grown in DMEM medium containing 10% Fetal Bovine Serum (BioSera). CHM13-hTERT cells (CHM13) [23] were cultured as in [24]: in DMEM:F12 medium containing 10% Fetal Bovine Serum (BioSera) supplemented with 1x Gutamax (ThermoFisher—35050061), 1xNEAA (ThermoFisher 11140050), 1mM Sodium Pyruvate, 1x Insulin-Transferrin-Selenium (ThermoFisher—41400045).

### Purification of telomere-digesting chimeric endonuclease (T-EN)

The telomere-digesting TRAS1EN-TRF1 chimeric endonuclease (T-EN) was expressed from a pET21b plasmid kindly provided by H. Fujiwara (University of Tokyo) [21]. Briefly, histidine-tagged T-EN was expressed in BL21-CodonPlus-RIL competent cells at 20°C and purified by affinity chromatography on a 5 ml His-Trap FF crude column (GE Healthcare), the protein was further purified by gel filtration using a HiLoad Superdex 200 16/600 column (GE Healthcare).

### qPCR, dot blot and Southern Blot

qPCR was performed using the LightCycler 480 (Roche) system with previously described primer pairs specific for alpha satellite DNA, as target (5’-TCCAACGAAGGCCACAAGA-3’ and 5’-TCATTCCCACAAACTGCGTTG-3’) and for the 18S rDNA, as reference (5′-CTCAACACGGGAAACCTCAC-3 and 5′-CGCTCCACCAACTAAGAACG-3′). Fold enrichment was calculated with the ΔΔCt method as enrichment of the target sequence over the reference. For the dot blot experiments, 50, 100 and 200 ng of DNA from each fraction and from unfractionated genomic DNA were blotted on a membrane (Amersham Hybond -N+, GE Healthcare) using a BioDot apparatus (Bio-rad). Membranes were hybridized overnight at 42°C with digoxigenin-3’-labeled oligos as probes specific for CENP-B boxes (5’- ATTCGTTGGAAACGGGA -3’), Alu repeats (5’- ATACAAAAATTAGCCGGGCG -3’) or telomeres (5’- TAACCCTAACCCTAACCCTAACCCTAA -3’). Signal detection was performed with CDP Star solution (Roche) and imaged with a Chemidoc imaging system (Biorad).

For Southern blot analysis, 1:1000 of each fraction together with 300 ng of unfractionated, digested gDNA were loaded on a 0.8% agarose gel in 0.5X TBE. Electrophoresis was performed at 5 V/cm for 90 minutes. After depurination, denaturation and neutralization, the DNA was blotted by capillarity on an Amersham Hybond-X (GE healthcare) membrane and crosslinked in a UV Stratalinker 1800 (Stratagene) with 1200 J of 254 nm UV. The membrane was pre-hybridized 1 hour at 65° in Church mix (500 mM NaPi pH 7.2, 1 mM EDTA pH 8.0, 7% SDS, 1% BSA). Hybridization occurred overnight in Church mix with a telomeric TTAGGG probe [18] or centromeric probe (produced as described below). After three washes in Church wash buffer (40 mM NaPi pH 7.2, 1 mM EDTA pH 8.0, 1% SDS), radioactive signal was impressed on a FUJIFILM Storage Phosphor screen for 5 hours and acquired with Typhon Trio (GE healthcare).

Centromeric probe for Southern was produced by apha-^32^P-dCTP-labelling (Prime-a-Gene Labeling System, Promega cat #U1100) of a ~300 bp PCR product obtained with primers 5′-CAGAAACTTCTTTGTGATGTGTGC-3′ and 5’-GTTTTTATGGGAAGATATTTCCT-3’ on a template of human genomic DNA.

### Libraries preparation and sequencing

Illumina sequencing libraries were prepared from unselected genomic DNA (WGS) and from the same fractions F2, F3 and F4 that were analyzed by Southern blot. After shearing to an average fragment size of 250 bp with a Covaris ME220 Sonicator, libraries were prepared with Kapa Hyper Prep kit (Roche) according to the manufacturer’s instructions with 12 amplification cycles then they were sequenced on an Illumina NovaSeq 6000 using paired-end 100x100 as sequencing mode.

Nanopore sequencing was performed from fractions derived from an independent digestion and sucrose gradient experiment. Before preparation of libraries for Nanopore sequencing, fractions F4 to F6 were pooled and 9 μg of this DNA was treated with Short Read Eliminator kit (cutoff <25 kb, Circulomics cat# SKUSS-100-101-01) to further remove contamination from shorter DNA fragments. Libraries were prepared from this sample, from fraction F3 and from total genomic DNA (WGS) using the Library Preparation by Sequencing kit (Oxford Nanopore Technology). For all samples, sequencing was performed on a Spot-ON Flow Cell (R9.4.1) on a MinION Mk1B device.

Libraries were quantified with Qubit dsDNA HS Assay Kit (Thermo Fisher) and checked by capillary electrophoresis with a TapeStation 4150 system (Agilent).

### Bioinformatic analysis

#### *In silico* digestion

The reference genome used is the T2T-CHM13v1.0, where the centromeric and non-centromeric regions were defined according to the ranges reported in S1 Table. The coordinates of satellite arrays belonging to the families HSat were defined based on the coordinates provided on the T2T-CHM13v1.0 reference and there named HSat1, HSat2, HSat3, HSat4, HSat5 (Altemose et al, 2021a). Only arrays longer than 5 Kb were selected and used for the *in silico* digestion (S2 Table). *In silico* digestion was performed by matching the occurrence of each restriction site sequence and replacing it with a line break. The lengths of the resulting strings were used to represent the size of digestion products. Distribution analysis and plotting was performed with RStudio [25].

#### Illumina sequencing

Illumina reads from all the fractions and from WGS were downsampled to the same total read count. The estimate quantification of α-satellite-derived Illumina reads was performed by counting the reads containing at least two of the previously identified unique alpha 18-mers representative of the alpha satellite DNA variation in the human genome [26]. To identify active HORs on chr15, previously published CENP-A CUT&RUN-seq reads [27] (NCBI accession number: PRJNA546288) were re-mapped on the new reference assembly as reported below.

All Illumina reads were mapped using bwa-mem algorithm of the BWA software package [28, 29] on the Telomere-to-Telomere T2T-CHM13v1.0 reference genome [30]. Reads mapping on centromeric regions were counted according to the ranges specified in the S1 Table. Reads mapping on different families of repeats were counted according to the ranges defined by the track Repeat MaskerV2 (http://t2t.gi.ucsc.edu/chm13/hub/t2t-chm13-v1.0/rmskV2/rmskV2.bigBed) retrieved by UCSC Table Browser [31] on the assembly T2T-CHM13v1.0. Enrichment and CUT&RUN-seq profiles were generated with deeptools 3.1.0 bamCompare [32] with a bin size of 2 Kb. Enrichment domains were defined as the regions where fold enrichment compared to WGS is higher than 5-fold. The overlap between enrichment domain and centromeric regions or HOR arrays was determined with bedtools intersect (version 2.21.0) [33].

To measure the fraction of enrichment domains comprised in HOR arrays and to plot HOR positions in figures, the coordinates of non-divergent HORs on T2T-CHM13v1.0 were used, as defined in [34], and as reported in S3 Table.

#### Nanopore sequencing

Nanopore sequencing data was basecalled with Guppy version 4.0 with a high accuracy model (*dna_r9*.*4*.*1_450bps_hac*.*cfg*) and were mapped using Winnowmap 2.0 [35, 36]. Primary alignments were filtered using samtools (version 1.9, [37]) option -F 2308. Samtools mpileup was used to quantify the number of mapped bases within centromeric regions (S1 Table).

#### Methylation analysis

Methylation analysis of nanopore data was performed as in [4]. Briefly, from the nanopore data mapped with Winnowmap 2.0 [35, 36], reads mapping in the centromeric regions were extracted and processed by nanopolish call-methylation tool (version 0.13.2, [38]), which extracts methylation information taking into consideration the raw nanopore current.

We filtered methylation calls using the *nanopore_methylation_utilities* tool [39] and generated a methylation frequency, that was used for extracting coverage on methylation data. The methylation frequency of each site is calculated as the number of reads where the site is called as methylated over the total number of reads where that site has any valid call (either methylated or unmethylated). Reads where methylation is not called are excluded. IGV [40] was used to visualize most of the data.

The donut chart reporting changes in the methylation status was generated from the CpG sites whose methylation is called in both WT and KO samples. When the variation in methylation was within the ±10% range, CpG sites were counted as unchanged; when the change in methylation (KO compared to WT) was higher than +10% or lower than -10%, then the site was counted as increased or decreased, respectively. Only sites where methylation level is > 40% in WT were considered.

### Combined Bisulfite Restriction Analysis (COBRA)

Genomic DNA (1 μg) was bisulfite converted using the EpiTect bisulfite kit (Qiagen) according to the manufacturer’s protocol. Converted DNA was amplified by PCR using Platinum Taq DNA polymerase (Invitrogen) with locus-specific primers described in Velasco *et al*, 2018 [41]. The PCR products were then digested for 3 hours with 10 U of BstBI (NEB) at 65°C for HSATII, HpyCH4IV (NEB) at 37°C for α-sat and LINE1, and BstUI (NEB) at 60°C for MAEL. An equal amount of PCR product was used for the undigested control and loaded in 3% agarose gel. Images were acquired using a ChemiDoc (BioRad) and the proportion of methylated (digested products, lower bands) versus unmethylated DNA (undigested product, upper bands) was quantified using Fiji.

### DNA combing

Combing and FISH analysis was performed on genomic undigested DNA and on the pool of fractions F4 to F6 from the SNE digestion. DNA was diluted in 0.25 M MES buffer (pH 5.5) and the DNA/MES mix was combed onto silanized coverslips (Genomic Vision) using the Molecular Combing System (Genomic Vision). DNA fibers were denatured for 5 min in 1N NaOH, followed by PBS (4°C) wash and dehydration in increasing concentrations of ethanol (75, 85, and 100%). Slides were hybridized overnight at 37°C with a biotinylated RNA α-satellite probe [see [42]] and washed 3 times with 50% formamide solution at RT. After 3 washes in 2X SSC and a quick wash in PBS, slides were incubated for 1h in blocking solution (blocking reagent Roche, 11096176001) at 37°C. Centromere signal was detected by alternating layers of avidin FITC (1:100, 434411, Thermo) and goat anti-avidin biotin conjugated (1:50, BA-0300-.5, EuroBioSciences) antibodies. Single stranded DNA was detected with rabbit anti single-stranded DNA antibody (1:2, JP18731, Tecan/IBL international) and anti-rabbit Cy^TM^3 (1:250, 711-165-152, Jackson Immuno Research). Fibers were mounted in ProLong Gold antifade reagent (P36935, Invitrogen) and acquisition was performed with an epifluorescence microscope (Upright ZEISS Apotome). To measure replication, cells were labeled in culture for 20 min with 5-Chloro-2-deoxyuridine (10μM, C 6851, MERCK) either in absence or presence of 16 hours Aphidicolin (100 nM, A-0781, Sigma Aldrich). Replication tracts were detected by Rat anti BrdU (1:25, ab6326, Abcam) and Goat anti Rat IgG (H+L) Alexa Fluor 594 (1:50, 712-585-150, Jackson Immuno Research).

## Verification and comparison

### *In silico* digestion of a human reference genome

Taking advantage of recent progress in the determination of the sequence of human centromeres, we performed *in silico* digestions of the T2T-CHM13v1.0 reference genome [6] using restriction sites from a panel of 240 commercially available restriction enzymes. We then verified the size distribution of fragments deriving from either centromeric or non-centromeric regions. Based on this analysis we identified 2 candidate enzyme combinations (ScrFI + NlaIV + EcoO109I and ScrFI + EcoO109I + BstUI, hereafter named SNE and SEB respectively) that are predicted to cut non-centromeric DNA at high rate, while digesting the centromeric regions at low frequency (**S1A and S1B Fig**). In both combinations, about half of centromeric DNA is digested into low molecular weight (LMW) fragments (**S1A and S1B Fig**). However, with the SNE combination a high level of enrichment in centromeric DNA fragments is predicted in the HMW range (up to >80% of centromeric fragments >55 kb) (**Fig 1B**) corresponding to an abundance of centromeric base-pairs up to 60% (**Fig 1C**). The SEB combination also showed a high percentage of centromeric fragments (40 to 60%) and centromeric base-pairs, but more homogeneously distributed in the range >15 kb (**Figs 1C** and **S1C**). Considering that in the reference genome the centromere content is about 2.8%, both combinations reach an enrichment in base-pairs of >20-fold.

### Centromeric DNA purification from human cells

**Fig 2:**
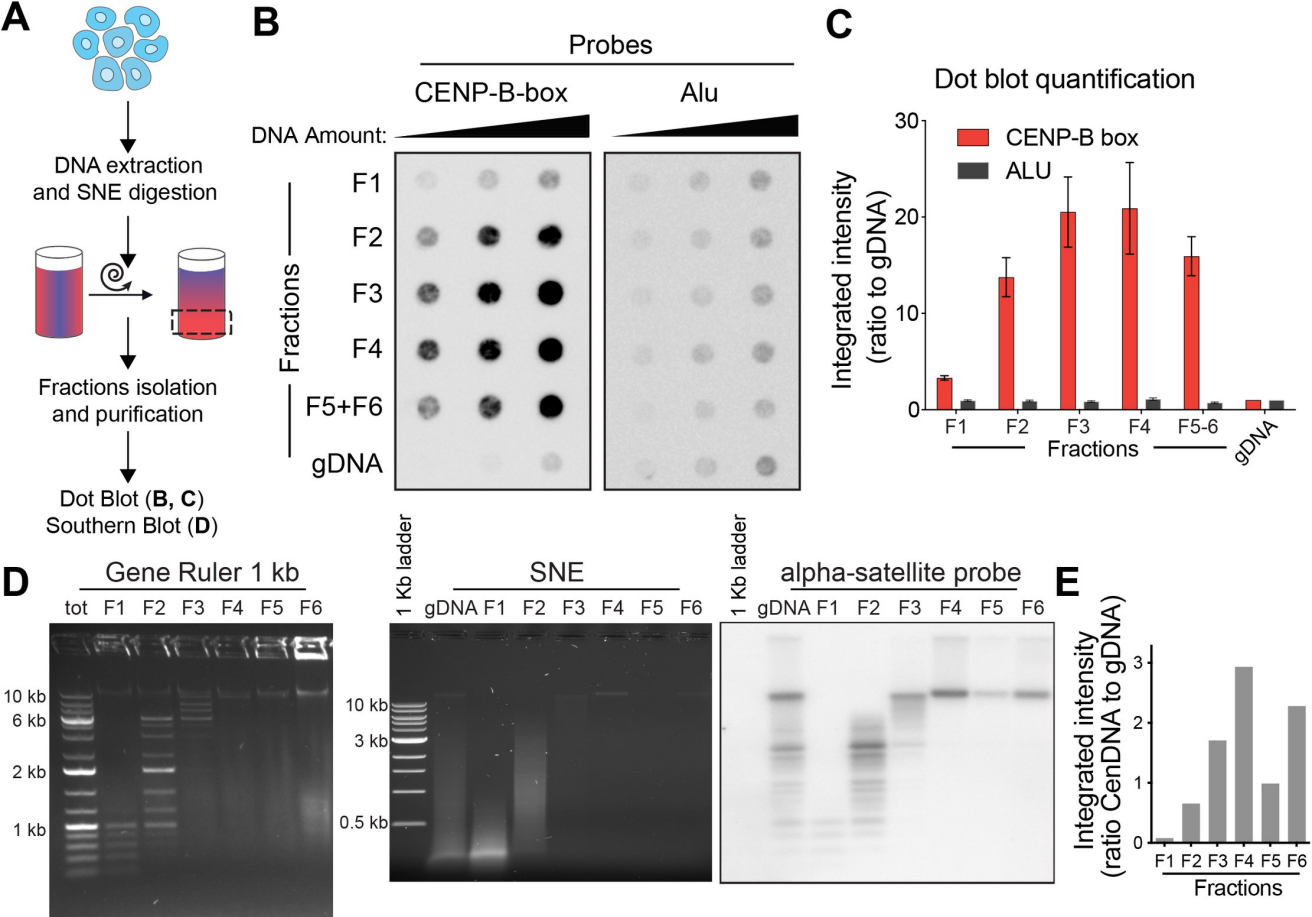
Centromeric DNA is enriched in the high-molecular weight fractions. **A**. Schematic representation of the experimental design. **B.** Dot-blot detecting the abundance of centromeric DNA (measured by signal intensity with a CENP-B box DNA probe, left membrane) in different sucrose gradient fractions (F1 to F4; F5+F6 is a pool of fractions F5 and F6) and in unfractionated genomic DNA (gDNA). A probe for the Alu repeat was used as a control (right membrane). In both membranes increasing amounts of DNA were loaded (50, 100 and 200 ng). **C**. Quantification of the dot-blot showed in B; signal is reported as a ratio to gDNA. The average for the different amounts of DNA is reported. Error bars represent the standard error of the three DNA amounts. **D**. Left: agarose gel electrophoresis performed on a molecular weight marker (Gene Ruler 1 kb), separated in the sucrose gradient showing efficient size separation; “tot” represents the unfractionated marker and F1 to F6 represent the different fractions. Middle and right: agarose gel electrophoresis of the sucrose fractions of a genomic DNA sample digested with the SNE combination and corresponding Southern blot after hybridization with an alpha satellite probe. “gDNA” represents the digested unfractionated sample and F1 to F6 represent different fractions. Lambda DNA digested with HindIII was also used as size control. **E.** Bar graph showing the ratio between CenDNA (from the Southern blot) over total DNA (from the agarose gel electrophoresis) in the fractions F1-F6.

To test these predictions, we extracted and digested DNA from a pseudo-diploid, colorectal cancer cell line (DLD-1) with the SNE enzyme combination. The digested DNA underwent size fractionation by sucrose-gradient ultracentrifugation (20% to 40% sucrose weight/volume) and the collected fractions were used for dot-blot hybridization with a centromeric probe (CENP-B box) (**Fig 2A**). As a control we used a probe targeting the Alu repeats, an element which is widespread across the genome and not disproportionately abundant at centromeres. Indeed, short (250–300 bp) Alu sequences occupy about 307,000 kb of the genome (~11%) [43, 44], but less than 20 elements per Mb are present at centromeres, and only within divergent alpha satellite [34]. Compared to unfractionated genomic DNA (gDNA), fractions 3 and 4 show the highest level of enrichment in centromeric DNA (about 20-fold), while Alu repeats were homogenously distributed (**Fig 2B and 2C**). The abundance of centromeric sequences in these fractions was also confirmed by qPCR for both the SNE and the SEB combinations (**S2A Fig**), further proving that the candidate enzyme mixes can be combined with size fractionation to enrich in centromeric DNA.

Restriction-based enrichment methods have been successfully used for telomeres since telomeric repeats do not contain restriction sites (including the recognition sites of our selected enzymes), therefore carryover of telomeric DNA may result in a decrease in the desired enrichment in centromeric DNA. To verify whether the centromere-enriched fraction also contained high amounts of telomeric DNA, we digested another batch of DLD-1 genomic DNA with the SNE enzyme combination, with the addition of a purified TRAS1EN-TRF1 fusion protein (T-EN), capable of cutting within telomeric repeats [21]. Following hybridization with centromeric or telomeric probes, we observed that while telomeric DNA is also detected mostly in the fractions F3 and F4, centromeric DNA still appears to be dominant (as expected due to its abundance over telomeric DNA in the human genome). Addition of T-EN successfully depletes most of the telomeric signal (**S2B and S2C Fig**), suggesting that it can be used in centromere enrichment from cell lines characterized by very long telomeres (e.g. ALT cell lines as U-2 OS).

To obtain information on the size distribution of the fragments resulting from digestion and fractionation, genomic DNA from diploid, non-transformed human hTERT RPE-1 cells was digested with SNE or SEB and analyzed by Southern blot using an α-sat specific probe (**Figs 2D** and **S2D**). While the bulk of digested DNA is in fraction F1 and F2 (visualized as a smear in the agarose gel) and almost invisible in the HMW fractions (F3 to F6), centromeric signal is detected mostly in fractions F3 and F6 when compared to the intensity of total DNA (on the agarose gel) (**Fig 2D and 2E**). Although this type of gel does not allow high resolution in the HMW range, fractions F4-6 appear to be >10 kb long, which makes them suitable for approaches requiring long DNA molecules, such as long-read sequencing or direct visualization by electron microscopy. As predicted *in silico*, some centromeric DNA is also detected in LMW fractions (F1 and F2), indicating that about half of centromeric DNA is digested into shorter fragments. Similar results were obtained for the SEB combination (**S2D Fig**). Hybridization with a telomeric probe shows that most of the telomeric DNA remains in F2, and HMW fractions (F4-6) are nearly devoid of telomeric repeats (**S2E Fig**) while being rich in centromeric DNA. Southern blot analysis on another replicate of RPE-1 cells and on different cell lines (DLD-1 cells, CHM13 and two different genetic backgrounds of HCT116 cells) revealed a nearly identical pattern of enrichment in the HMW fractions when hybridized with an α-sat probe (**S3 Fig**). These results highlight the reproducibility of our digestion and size fractionation approach on the distribution of centromeric fragments across different cell types.

### Assessment of centromeric DNA enrichment by Illumina DNA sequencing

**Fig 3:**
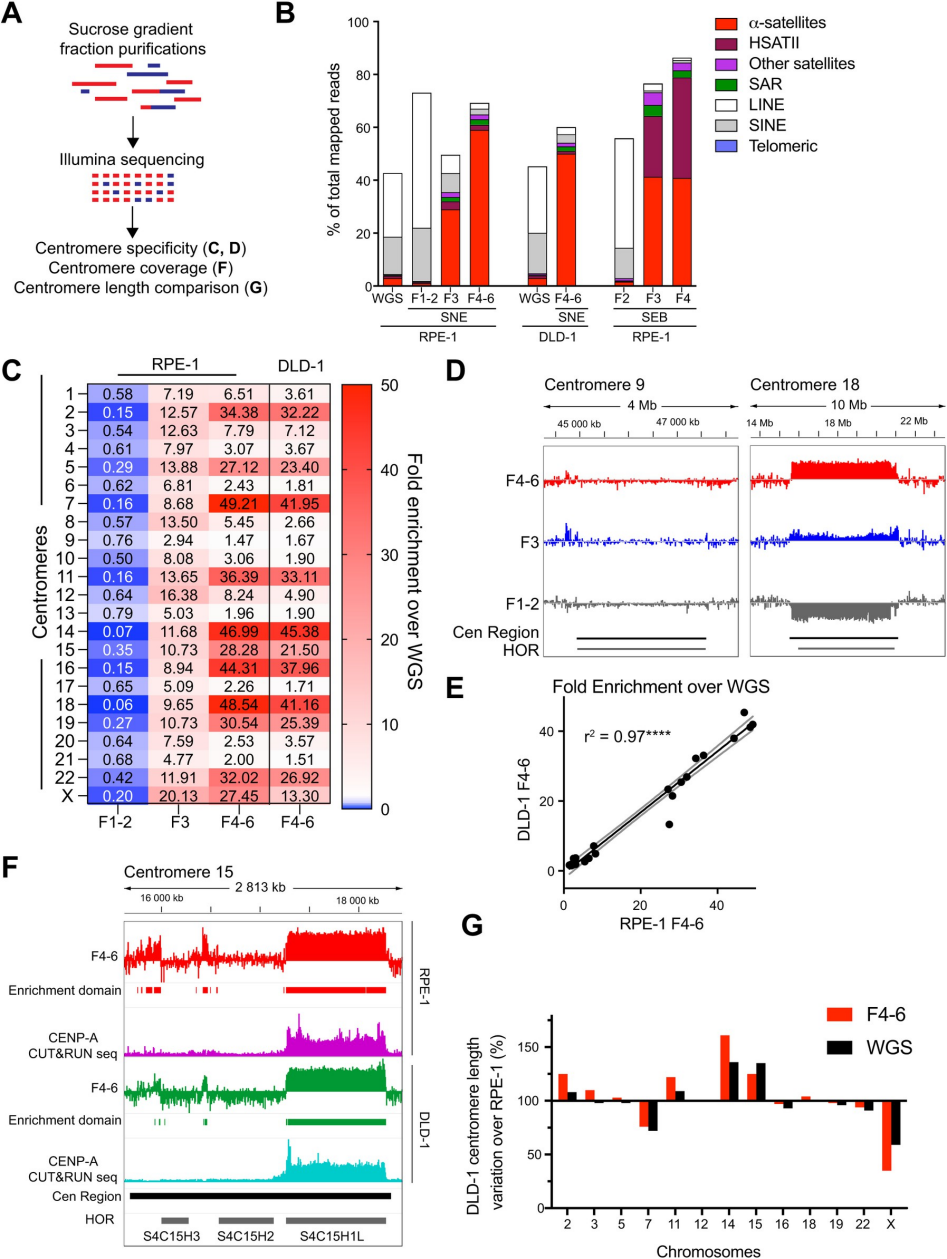
The CenRICH method provides high enrichment of alpha satellite and HSAT II DNA. **A**. Schematic representation of the experimental design. **B**. Quantification of Illumina reads mapping on centromeric regions (red) and on other families of repetitive DNA after CenRICH (digestion with SNE or SEB enzyme combinations) and in an undigested unfractionated sample (WGS). F1-2 represents a pool of fractions 1 and 2 (LMW), F4-6 represents a pool of fractions from 4 to 6 (HMW). Data from RPE-1 (SNE and SEB) and DLD-1 (SNE only) are shown. Read counts are reported as a percentage of total mapped reads. **C**. Enrichment in centromere-derived reads after Illumina sequencing across the different centromeres in fractions F1-2, F3 and F4-6 (for RPE-1 cells) and fraction F4-6 (for DLD-1) after CenRICH with SNE digestion. Enrichment is expressed as a ratio to the read counts in the corresponding WGS samples. **D**. Examples of enrichment profiles in different fractions (F1-2, F3, and F4-6) after SNE digestion and sucrose gradient fractionation of RPE-1 DNA. On the left panel, centromere of chromosome 9 does not show enrichment in any fraction. On the right panel, centromere 18 shows high enrichment in F4-6 and depletion in fractions F1-2. Enrichment is plotted as log2 ratio over WGS in 2-Kb wide genomic bins. Y-axis ranges between -8 and +8. Genomic coordinates on the T2T-CHM13v1.0 reference are reported on top. Boundaries of centromeric regions (Cen Region, black bars) and HORs (grey bars) are described in S1 and S3 Tables, respectively. **E.** Scatter plot and linear regression reporting the correlation in fold enrichment (ratio to WGS) between the F4-6 fractions of RPE-1 and DLD-1 cells (SNE digestion, Illumina sequencing). Each of the 23 dots represents a centromere. The dashed line represents 95% confidence intervals of the linear regression. R-square = 0.97, p-value <0.0001. **F.** Example of enrichment profile and identification of enrichment domains on centromere 15, for fractions F4-6 after CenRICH with SNE enzyme combination on RPE-1 (red) and DLD-1 (green) cells. Enrichment is plotted as log2 ratio compared to WGS along 2-Kb bins (y-axis range -4 to +6). Bars below the enrichment profile identify enrichment domains where fold-enrichment is > 5-fold. Purple and cyan profiles report CENP-A CUT&RUN-seq profiles as ratio to WGS, identifying the enrichment domain as corresponding to the active HOR (y-axis range from 0 to 15). Centromeric region (Cen region, black bar) and HOR boundaries (grey bar) are defined in S2 and S3 Tables. **G.** Estimation of the variation in centromere length in DLD-1 cells compared to RPE-1, calculated from WGS or from F4-6 after CenRICH with SNE enzyme combination. Y-axis reports the percentage variation in the number of reads mapping in centromeric regions (DLD-1 over RPE-1), which is used as a proxy for centromere length.

Next, we repeated the enrichment protocol on RPE-1 cells with the SNE enzyme combination and sequenced with an Illumina NovaSeq 6000 system all the DNA fractions (**Fig 3A**). We pooled the DNA with similar size ranges (as deduced by the Southern blots, **Figs 2D** and **S2E**): LMW fractions (F1-2), F3 and HMW fractions (F4-6). We used unfractionated DNA sample as control (hereafter referred as WGS). We also included HMW fractions (F4-6) from a DLD-1 cell line for enrichment comparison. The resulting reads were then mapped on the T2T genome (T2T-CHM13v1.0 [30]). The reads were counted as centromeric when aligning within the genomic coordinates (reported in **S1 Table**) that contain both homogeneous HORs and monomeric/divergent α-sat, hereafter defined together as “centromeric regions”. In RPE-1 cells, about 59% of the reads from F4-6 fractions map on centromeric regions, while only ~2.9% of F1-2 and WGS reads are centromeric (**Fig 3B**). This corresponds to an approximately 20-fold enrichment in centromeric DNA compared to WGS. Very similar results were obtained for DLD-1 cells in the HMW fractions (**Fig 3B**).

The mapped reads were then further analyzed to test for the presence of other repetitive DNA families using RepeatMaskerV2 annotations: fractions F3 and F4-6 in RPE-1 and DLD-1 digested with SNE combination led to about 10–12% of reads mapping on other satellite DNA (**Fig 3B**), notably belonging to the families of satellite II (HSATII) and SAR (more recently recategorized as HSat1A) [34]. As expected, short interspersed mobile elements like SINEs and LINEs are underrepresented in the high molecular weight fractions and tend to remain in the F1-2 fractions (**Fig 3B**). Only very low levels of telomeric DNA were identified, mainly in F1-2, as expected from RPE-1 cells and from the Southern blot results (**S2E Fig**). Interestingly, performing CenRICH with the SEB digestion mix results in a much higher abundance of the pericentric HSATII (23% and 38% for F3 and F4, respectively, with an enrichment up to 41-fold compared to WGS), with only a minor decrease in the fraction of centromeric DNA (**Fig 3B**).

To verify efficiency of the restriction digestion, the Illumina sequencing data were tested for the presence of restriction sites within the reads, indicative of an incomplete digestion. In the centromeric enrichment fractions of the SNE combination we detected very low level of intact restriction sites (<5% of total sites observed by WGS), indicative of a near-complete digestion efficiency (**S4A Fig**). Although ScrFI and NlaIV are CpG DNA methylation-sensitive enzymes, their digestion rates are extremely high, suggesting that most of these sites are unmethylated. In the SEB combination we detected a slightly higher fraction of undigested sites for the BstUI enzyme (15% and 10%) (**S4A Fig**), possibly due to the increased effect of DNA methylation protection for this restriction site that contains two CpG dinucleotides.

To avoid the influence of potential mapping artifacts, we performed a k-mer based analysis aimed at identifying the reads containing α-sat sequence, while not relying on alignment to a reference assembly (see Methods). 45–50% of reads were identified as alpha satellite in the SNE sample (**S4B Fig**), a value that is compatible with the ~59% of reads mapping within centromeric regions, where not all DNA is α-sat (for example, transposable elements are present within arrays of divergent alpha repeats at a frequency of >90 transposable elements per Mb [34]).

We then verified if centromere-derived reads in the enriched fractions are homogeneously distributed across chromosomes or if some centromeres are more represented than other. Centromeres of different chromosomes are characterized by different HORs on which reads can be differentially mapped thanks to the recent improvement in the assembly of human centromeres [4, 5, 30]. Analysis on the HMW fractions reveals that the distribution of the centromeric reads is heterogeneous, with some centromeres being largely overrepresented (e.g.: ~49-fold enrichment for centromere 7 in RPE-1 F4-6) compared to the undigested, not fractionated WGS (**Figs 3C**, **3D** and **S4C**). Overall, 21 out of 23 centromeres are enriched by at least 5-fold in either F3 or F4-6 of the RPE-1, while only chromosomes 9 and 13 show < 2-fold enrichment in F4-6. Comparison between the centromeric specific fold enrichment in RPE-1 and DLD-1 HMW fractions showed a high degree of correlation (**Fig 3E**, r^2^ = 0.97), reinforcing the reproducibility of our centromeric enrichment method. Performing the same analysis on the fraction F4 of the SEB digestion also shows inter chromosomal heterogeneity, but with a different pattern of centromeric reads distribution compared to SNE (**S4D Fig**). As expected, in the LWM fractions (F1-2), centromeric sequences are underrepresented compared to WGS, consistent with their higher abundance in other fractions and tend to be inversely proportional to the enrichment in HMW fractions (**Figs 3D** and **S4C**). It is important to point out that during Illumina library preparation, fragments below 200 bp are excluded: therefore, it is possible that highly fragmented centromeric molecules will not be represented in any of the fractions we sequenced.

The estimation of fold enrichment is informative to understand abundance of reads mapping on each centromere, but it does not allow to understand how the enrichment is distributed along the α-sat array. To elucidate this, we defined discrete domains where a fold enrichment of at least 5-fold is detected by counting the reads in WGS and F4-6 mapping in 2 kb bins. We then measured the proportion of each centromeric region (as defined on the T2T reference genome) that is included within these enrichment domains. Only centromeres that show at least 5-fold enrichment in F4-6 were analyzed (**Fig 3C**). Our results indicate that the entire centromeric region is not fully represented in the enriched fraction, but most centromeres maintain more than 60% of the cumulative HOR arrays length (**S4E Fig**). Some centromeres are almost fully included (e.g. centromere 14: 2.35 out of 2.72 Mb) and others only partially (e.g. centromere 15, 1.1 out of 2.64 Mb). If we exclude monomeric/divergent alpha satellite and limit the analysis to HOR arrays (according to coordinates reported in S2 and **S3 Tables**, see material and methods), these proportions tend to be higher (**S4E Fig**). This is in agreement with the notion that α-sat organized in HOR is more homogeneous [7] and sequence variations leading to the appearance of restriction sites are more likely to occur in divergent/monomeric α-sat. Moreover, when different HOR arrays are present in the same centromere, our enrichment can preferentially over/under-represent specific HOR arrays: for example, on centromere 15 only one out of three HOR arrays is highly enriched (D15Z3, recently renamed S2C15H1L), due to high frequency of restriction sites on the other two HOR arrays (**Fig 3F**). This HOR was found to be the one carrying centromere activity (CENP-A binding) in CHM13 [7], and RPE-1 and DLD-1 cell lines [27] (**Fig 3F**). Consistently with our data on fold enrichment, comparison between DLD-1 and RPE-1 shows high level of correlation in the proportion of HORs that are included in the enrichment (**S4F Fig**).

Previously, Illumina sequencing was used to estimate the length of human centromeres in both RPE-1 and DLD-1 cells [27], highlighting some differences between these two cell lines. Given the homogeneous nature of alpha satellite HORs, the principle of this length estimation is that longer alpha satellite arrays will originate more reads, resulting in a proportionality between centromere length and the number of reads. We tested if the enriched F4-6 sample can be used as a substitute of WGS to compare the relative sizes of specific centromeres: we compared centromere lengths between DLD-1 and RPE-1 cell lines by counting the number of reads mapping on centromeric regions of the T2T CHM13v1.0 assembly. By plotting the variation in centromeric read counts (as a proxy of length) for centromeres with at least 5-fold enrichment in DLD-1 compared to RPE-1, we observed a similar trend in F4-6 and WGS: centromeres detected as longer or shorter in WGS show also an increase or decrease in F4-6 (e.g. Cen14, Cen15; **Fig 3G**). As expected, since DLD-1 is a male cell line while RPE-1 is female, both WGS and F4-6 data show a decrease in the abundance of CenX-derived reads.

In conclusion, while CenRICH sequencing cannot substitute WGS for *de novo* assembly of centromeres or comparison between different HORs in the same cell line (see discussion), this method can provide crucial information on the direction of the variation in the length of specific centromeres across different cell lines or experimental conditions. In these settings, the 20-fold enrichment in Illumina reads obtained with CenRICH allows a higher coverage than WGS at centromeric regions (**S4 Table**), thus reducing the impact of sequencing errors and potentially improving the detection of some sequence variations (see discussion).

## Applications

### Using CenRICH to study DNA methylation with Nanopore sequencing and DNA replication with DNA combing

**Fig 4:**
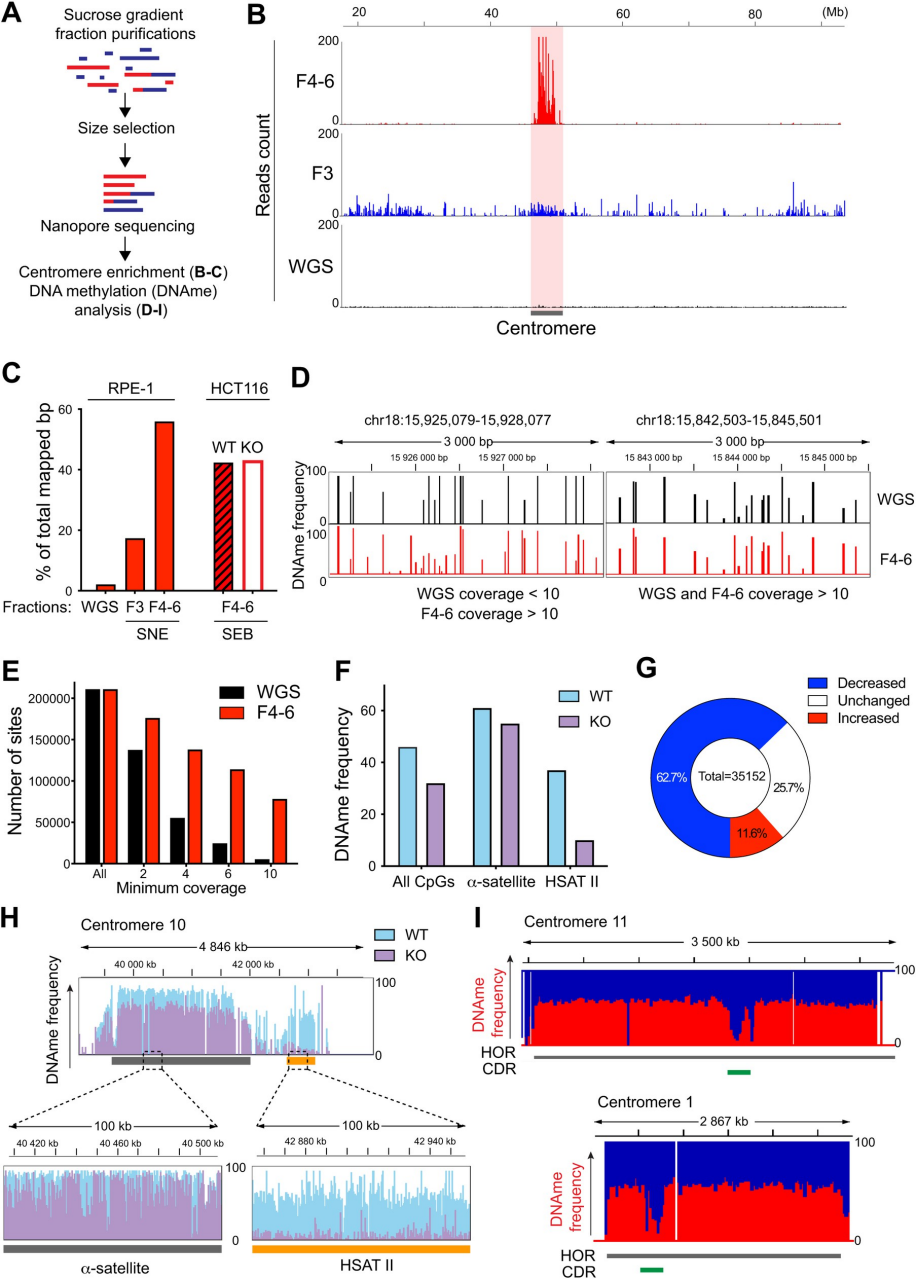
CenRICH is suitable for long read sequencing and DNA methylation analysis. **A**. Schematic representation of the experimental design. **B.** Coverage profiles of the centromeric region of chromosome 5 after Nanopore sequencing of an undigested sample (WGS), fraction 3 (F3) and a pool of fractions F4, F5 and F6 (F4-6) after CenRICH with SNE enzyme combination. Genomic coordinates are reported on top. **C.** Quantification of base-pairs from Nanopore reads that map within the centromeric regions (as defined in S1 Table) after CenRICH with the SNE enzyme combination (RPE-1 cells, fractions F3 or a pool of fractions F4 to F6) or SEB enzyme combination (HCT116 cells, pool of fraction F4 to F6). WT and KO indicate the genotype of the two HCT116: wild-type or DNMT1 and DNMT3B knock-out, respectively. WGS indicates the value for an undigested unfractionated sample of RPE-1 DNA. Base-pair counts are expressed as percentage of total number of mapped base-pairs. **D.** Methylation frequencies of individual CpG sites (expressed as a level from 0 to 100%) detected by Nanopore sequencing in different regions of centromere 18. WGS represents a non-enriched sample of RPE-1 cells, while F4-6 represents RPE-1 DNA that underwent CenRICH. Only CpG sites that are called both in WGS and F4-6 are reported. Left panel shows a portion of cen18 where methylation calls derive from a coverage of less than 10 reads in WGS but more than 10 in F4-6. Right panel shows a portion of cen18 where methylation calls derive from a coverage of more than 10 reads in both samples. **E.** Distribution of CpG sites based on the minimum coverage. The minimum coverage is expressed as number of Nanopore reads covering the site. WGS: undigested unfractionated RPE-1 sample. F4-6: fractions F4 to F6 after CenRICH with SNE enzyme combination on RPE-1 cells. **F.** Average methylation frequency (expressed as percentage) detected by Nanopore sequencing in HCT116 cells that underwent CenRICH with the SEB enzyme combination. Values are reported for all included CpGs, for the ones in alpha satellite and for the ones in HSATII. WT and KO indicate the genotype of the two HCT116: wild-type or DNMT1 and DNMT3B knock-out, respectively. Only CpGs with methylation calls in both samples are included in the analysis. **G.** Proportions of CpG sites that show higher, lower, or equal methylation frequency in HCT116 DNMT1/3B double KO (KO) compared to HCT116 wild-type (WT), same cell samples as in F. Only sites with methylation frequency > 40% in WT and coverage >10 in both samples are included. A site is labelled as increased or decreased if the difference in methylation frequency is respectively > 10% or < -10% in KO compared to WT, otherwise it is labelled as unchanged. **H**. Example of methylation frequency measured by Nanopore sequencing on the same HCT116 samples as in F. The genomic interval includes the alpha satellite array of chromosome 10 (grey bar) and a flanking pericentromeric HSATII array (yellow bar). Y-axis ranges from 0 to 100. **I.** Example of detection of Centromere Dip Region (CDR) on chromosomes 11 and 14 after performing CenRICH with the SNE combination on CHM13 cells. The red profile represents the methylation frequency with y-axis ranging from 0 to 100%. White portions of the plot represent regions with no methylation calling. The green bar represents the CDR as previously identified in CHM13 [34].

Since long-read sequencing techniques have become crucial for the dissection of repetitive arrays like centromeres, we tested the applicability of CenRICH to Nanopore sequencing, with particular interest in the centromere enrichment level and in DNA methylation (**Fig 4A**). Following DNA digestion with SNE and size fractionation on RPE-1 cells, sucrose gradients fractions F4 to F6 (F4-6) were pooled and sequenced with the Oxford Nanopore Technologies system. In parallel, fraction F3 and an undigested sample (WGS) were also sequenced. A capillary electrophoresis analysis showed that, while the mass of F4-6 consists mainly of fragments >50 kb, contamination with molecules down to ~1 kb is also present (**S5A Fig**, red line), which negatively affected the average read length in the output of Nanopore sequencing. Therefore, prior to further sequencing we used a size selective precipitation method (see “description of the method”) to efficiently remove DNA molecules <10 kb and to additionally enrich the sample in long DNA fragments (**S5A Fig**, blue line). Sequencing of this sample led to a N50 of ~22 kb (50% of the sequenced base-pairs are within reads >22 kb long) with about 40% of reads longer than 15 kb (**S5B Fig**). Following this additional step of size purification, for most centromeric regions there is a strikingly higher centromeric DNA abundance in F4-6 fractions compared to WGS. Specifically, F4-6 shows a >26-fold enrichment in overall centromeric DNA compared to WGS, with about 55% of the total sequenced base-pairs being of centromeric origin (**Fig 4B and 4C**). F3 has an enrichment level of ~8-fold, with 17.5% of the base-pairs deriving from centromeric regions. In summary, the restriction digestion-based centromere selection method can efficiently be used in combination with Nanopore sequencing to reach unprecedented levels of enrichment in centromeric DNA, while also preserving several kb long reads at a fraction of the cost of WGS.

One advantage of the Oxford Nanopore Technologies system is that DNA methylation can also be detected. To test if CenRICH can also be used to measure methylation of α-sat, we performed methylation calling on the sequencing outputs of WGS and F4-6 in RPE-1 cells. Starting from about 12 Gb of sequencing output from the WGS sample, we obtained methylation calls (measurement of methylation level ranging from 0 to 100%) for 278862 CpG sites located within the centromeric regions. Instead, the F4-6 sample showed more methylation calls (445911 centromeric sites) starting from only 1.9 Gb of sequencing data (**S5C Fig**), which is consistent with the enrichment in centromeric fragments observed in the HMW fractions. Taking into consideration the 211176 sites whose methylation is called in both samples, the average DNA methylation frequency of all CpGs is comparable (**S5D Fig**), suggesting that the enrichment procedure did not introduce a bias in the measurement of DNA methylation level. To further verify that the enrichment did not introduce distortions in the detection of methylation status, we performed a pairwise comparison of all CpG sites with at least a coverage of 10 in WGS and F4-6. We detected a high correlation with little to no CpG sites showing high methylation in WGS and low methylation in F4-6, and vice-versa (**S5E Fig**).

High sequencing coverage reduces the impact of sequencing errors and allows more accurate detection of sequence variations including mutational signatures, polymorphisms and base modifications. The accuracy of DNA methylation calling is also influenced by the coverage: when a CpG site of the genome is covered by several sequencing reads, the methylation call will be less likely to be affected by stochastic fluctuations and will more faithfully represent potential heterogeneity in the cell population or between homologous chromosomes. Incorporation of CenRICH has the potential to increase the quality of DNA methylation calls at certain centromeric regions in proportion to the total amount of sequenced base-pairs. This can be exemplified here: by selecting a region with coverage > 10 reads in both F4-6 and in WGS, the methylation level of individual CpG sites is very concordant (**Fig 4D**, right panel); on the other hand, when the coverage is too low, more differences are detected between WGS and F4-6 (**Fig 4D**, left panel), which are more likely due to noise and stochastic variation rather than actual changes in the DNA methylation status. Overall, a higher proportion of F4-6 sites has high coverage compared to WGS, with a 14-fold increase in centromeric sites covered by at least 10 reads (**Fig 4E**).

To assess the ability to detect changes in DNA methylation status of peri- and centromeric repeats with our method, we performed CenRICH using the SEB enzyme combination which also allows to enrich in HSATII repeats. We used HCT116 cells, either wild-type (hereafter called “WT”) or depleted for both DNMT1 and DNMT3B (hereafter named “KO”) [45], two of the DNA methyl transferases responsible for the establishment/maintenance of CpG methylation [46]. Despite the KO cells still carrying variable and significant residual DNA methyltransferase activity [47], comparison of WT and KO is a good validation of our methodology. Nanopore sequencing on the enriched fractions F4-6 showed similar levels in centromeric base-pairs for both (**Fig 4C**). Methylation analysis on these two HCT116 samples resulted in almost 7 millions of CpG sites called in each cell line, with an overlap of about 5 million CpG sites and with a similar fraction of sites with coverage higher than 10 (**S5F and S5G Fig**), highlighting the similarity in the accuracy of DNA methylation calling. On the enriched F4-6 fraction, average DNA methylation level drops from 44% in WT to 32% in KO (**Fig 4F).** A pairwise comparison of the methylation status of all CpG sites shows a tendency towards a decrease in methylation in KO compared to WT, with the linear regression trendline being skewed from the diagonal (**S5H Fig**). As expected, DNA methylation frequency of most sites in KO cells is lower, while some CpGs still maintain their methylation level or even show a small increase (**Fig 4G**).

Taking advantage of the SEB combination of enzymes that allows retention of most pericentromeric sites, we then compared the variation in methylation status between α-sat and HSATII. Interestingly, the KO cell line showed a much greater decrease in DNA methylation level at the pericentromeric HSATII (~73%) than at the α-sat whose methylation remain on average unaffected (~9.8%) (**Fig 4F and 4H**). We confirmed these results using a different method (COBRA), that estimates DNA methylation in a sequence specific manner, reinforcing the validity of our findings (**S5I and S5J Fig**). These results also highlight the complexity in the regulation of DNA methylation of the (peri)centromeres, where the depletion of DNMT1 and DNMT3B does not simply result in a homogeneous loss of DNA methylation.

A very fine dissection of the DNA methylation status at human centromeres was recently achieved in the CHM13 cell line [4, 7, 48]. Within active α-sat HORs a subdomain of decreased methylation (called Centromere Dip Region, CDR) was identified, surrounded by flanking regions with higher methylation level. Using the same CHM13 cell line, whose genome is exactly the one represented in the T2T-CHM13v1.0 reference, we tested if these results can be reproduced with the CenRICH method. With only 0.5 Gb of mapped Nanopore reads, we accurately detected at the same position the CDR on the centromeres that showed the highest enrichment (**Fig 4I**).

Overall, combining the results obtained in RPE-1, HCT116 and CHM13 cells, we show that CenRICH provides accurate information on DNA methylation of (peri)centromeres (including distribution of DNA methylation), without introduction of biases with respect to WGS. This approach will be particularly useful in comparing selected target regions in different experimental conditions (e.g., treatments or mutants, as we have shown for the DNMTs KOs), with higher coverage obtained from lower sequencing depth.

**Fig 5:**
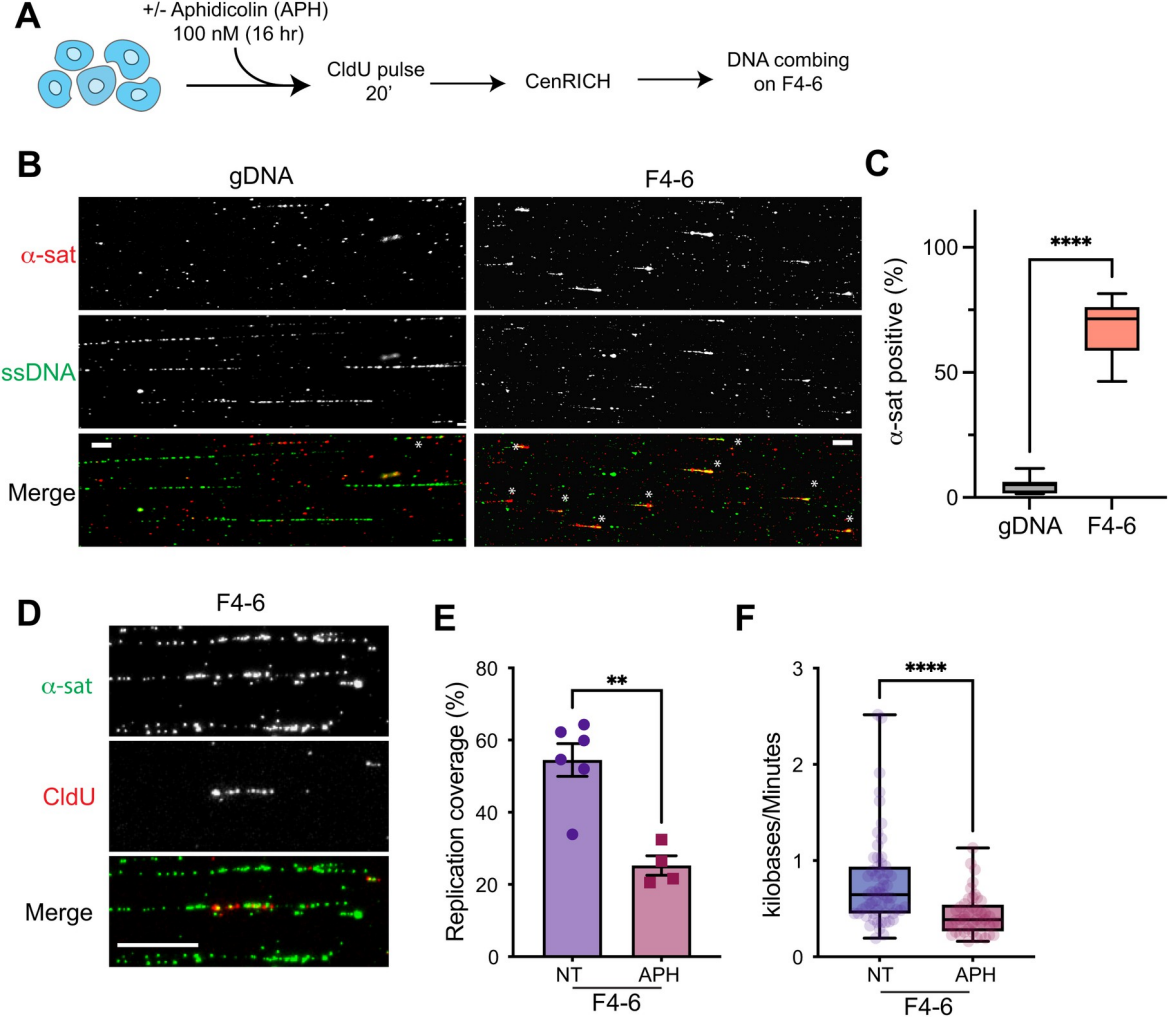
CenRICH is suitable for single molecule replication analysis. **A.** Schematic representation of the experimental design. **B.** Representative images of DNA fibers hybridized with an anti single-stranded DNA (ssDNA) antibody and biotin labelled α-satellite probe in the indicated condition. Asterisks mark α-satellite positive fibers. Scale bar 20 μm (gDNA) and 10 μm (F4-6). **C.** Box and whiskers plot shows the percentage of DNA fibers that are positive to hybridization with α-satellite probe, in a non-enriched sample of RPE-1 DNA (gDNA) or in the HMW fraction after CenRICH on RPE-1 cells with the SNE enzyme combination. Fibers of less than 10 kb were excluded from the analysis. n = > 350 fibers for F4-6 and > 230 for gDNA. Mann Whitney test, p < 0.0001. **D.** Representative images of CldU incorporation on combed DNA fibers, as marker of ongoing replication in RPE-1 cells that underwent CenRICH with the SNE enzyme combination. Scale bar 10 μm. **E.** Mean cumulative percentage of DNA fibers showing CldU incorporation in an untreated sample (NT) or in a sample treated with aphidicolin (APH). Both samples derive from RPE-1 cells after CenRICH with SNE enzyme combination. Each dot represents one image with an average of ~8 fibers. Error bars represent SEM. Mann Whitney test, p = 0.0095. **F**. Box and whiskers plot showing fork velocity (expressed as Kb/minute) as measured from the CldU incorporation rate. Same samples as E. Whiskers range from minimum to maximum values. Box is showing the 25, 50 and 75% percentile. n = 59 and 44 for NT and APH respectively. Mann Whitney test, p < 0.0001.

Finally, we tested the feasibility of single-molecule direct visualization on the centromere-enriched sample by fluorescence microscopy. To this end, DNA fibers from the pool of fractions F4 to F6 after SNE digestion (F4-6) of RPE-1 cells and undigested, unfractionated samples (gDNA) were subjected to DNA combing assay coupled with a mix of fluorescent probes against α-sat DNA (**Fig 5A**), as previously described [42]. Here we observed that in the pooled F4-6 sample the DNA fibers have a mean length of ~28 kb (median distribution of centromeric vs non-centromeric of 28 kb vs 22 kb, respectively; **S6A Fig**). In agreement with our DNA sequencing data, in the F4-6 sample about ~67% of DNA fibers > 30 kb are recognized by the α-sat DNA FISH probes, while only ~5% of the fibers are labelled in the gDNA sample (**Figs 5B, 5C** and **S6B**). This result indicates that CenRICH is suitable and facilitates single-molecule analysis of centromeric DNA fibers.

One of the main applications of DNA combing is the study of DNA replication speed. As proof of concept, we tested our capability of detecting variation in the replication speed at centromeric regions in the enriched sample. For this purpose, we performed a CldU incorporation assay on RPE-1 cells in untreated condition or following low dose of aphidicolin, a DNA polymerase inhibitor, to induce a mild decrease in replication speed without completely blocking replication [49]. While no change in the distribution of the fragment length between treated and untreated sample was observed (**S6C Fig**), the length of the CldU track (indicative of active replication) was on average shorter in the aphidicolin-treated sample (50% vs 25% average coverage of each fiber), consistent with a reduction of replication fork velocity (**Fig 5D–5F**).

In conclusion we show that CenRICH can be applied for the direct visualization of centromeric fragments by DNA combing. Our method also allows the study of variations in the replication dynamics of these loci, with the advantage of greatly increasing the amount of centromeric molecules in the sample.

## Discussion

In this manuscript, we provide a simple and reliable method to enrich for centromeric DNA, independently from the binding of proteins (unlike ChIP/Cut&Run on centromeric proteins) that we named CenRICH. We show that our approach is compatible with DNA sequencing (both short-reads and long-reads sequencers), preserves DNA methylation information, and is suitable for direct visualization of single DNA molecules.

The CenRICH method provides several advantages. First, it has great potential to make sequencing of centromeric DNA more affordable and efficient. From our results we can estimate that, to obtain an average coverage across centromeres of 15X, WGS would require sequencing of over 45Gb, while the enriched F4-6 would only need about 1.8 Gb, with a striking decrease in sequencing cost and time. Nanopore sequencing on a MinION or GridION device was recently estimated to have a cost ranging between 50 and 500 US$ per Gb [50]: using WGS to achieve 15X coverage at the centromeres would require between 2,250 and 22,250 US$. Taking the Nanopore data from the RPE-1 cell line, sequencing of a CenRICH sample to achieve a similar average coverage at centromeres would reduce this cost by 25-fold. This decrease in sequencing cost is largely enough to offset the additional expense required to perform the CenRICH procedure, estimated to be 220–320 € (approximately 235–340 US$) (**S5 Table**).

Second, for equal sequencing cost and amount of reads, CenRICH increases sequencing coverage at centromeres: with the >25-fold enrichment we obtained in Nanopore sequencing, a total of 15 Gb of sequencing output corresponds to an average coverage of 125X (with some regions reaching up to 245X) compared to just 5X that would theoretically be obtained with WGS. While the coverage is not equally distributed, the proportion of centromeric DNA with high coverage is drastically higher in CenRICH compared to WGS (starting from equal amount of Illumina reads) (**S4 Table**). Having more reads covering the same selected region reduces the impact of stochastic variation which may lead to lack of information in regions not covered by any reads and, also, improves the reliability of DNA methylation calling (**Fig 4D**). Our data on the comparison of HTC116 WT vs KO revealed that, upon depletion of two DNA methyl transferases, centromeric DNA tends to maintain its methylation status better than its surrounding pericentromeric regions (**Fig 4H**). This experiment provides an application of the CenRICH technique to tackle a biological problem, by suggesting differential methyl transferase recruitment mechanisms regulating the epigenetic landscape of α-sat DNA. On the same line, higher coverage can help with the detection of genetic variants (such as SNPs and other polymorphisms) and mutational signatures with greater confidence. An example of such application would be the identification of mutational signatures (e.g. base-pair substitutions, short insertions/deletions) at centromeres in different experimental settings. These could include analysis of APOBEC activities [51] that induce deamination and C to T substitutions or mismatch repair deficiencies.

Our data revealed heterogeneity in the level of enrichment obtained among different centromeres (**Figs 3C** and **S3D**). This observation highlights that different enzyme combinations or choice of fractions can be used to focus the enrichment on selected centromeres to further study their epigenetic status, length, and sequence variations. While the length of HOR arrays can vary between different individuals, their sequence tends to be homogeneous [52], with a low chance of the appearance or disappearance of several restriction sites that may significantly impact on the CenRICH approach. In agreement with this notion, our result on a limited number of cell lines show great correlation in centromere-specific enrichment levels (**Fig 3E**), suggesting that applying this enrichment method to other cell lines will give similar and predictable results in term of which centromeres will be more or less represented. While we have applied CenRICH to compare the lengths of specific HORs (**Fig 3G**) between two cell lines, we cannot exclude that more complex structural variations may be present in different individuals (e.g. presence of novel or rare HORs), leading to a different enrichment pattern from the one we observed. This possible source of variability may further be influenced by the reference genome in use, which may not fully represent the complexity of the α-sat arrays of certain individuals. Our method is not suitable to accurately estimate centromere length in base-pairs, as in some cases only part of the centromeric region is preserved (**Figs 3F** and **S4E**); similarly, this heterogeneity in enrichment (**Fig 3C**) does not allow to compare the length of different centromeres within the same cell line.

It is important to point out that WGS would still be the best approach for complete *de novo* assembly of entire α-sat arrays, since CenRICH does not guarantee a full recovery of all centromeric regions. Nonetheless, a combination of CenRICH and WGS may still benefit large projects of centromeric DNA sequencing and assembly by reducing costs and increasing coverage of certain regions.

A similar restriction-digestion method combined with agarose gel separation to enrich for centromeric DNA was recently developed [53]. Here the authors used a different enzyme combination (MscI and AseI) from the one presented here. *In silico* digestion with MscI and AseI revealed that centromeric DNA is less digested compared to SNE or SEB combination (**S6D Fig**), but overall, the percentage of centromeric fragments is lower compared to our enzyme combinations (**S6E Fig**), likely due to better preservation of non-centromeric DNA. While the *in silico* prediction can vary significantly from what is really observed in cells, the MscI-AseI combination represents a valid alternative to the one presented here when preservation of total centromeric DNA, but not its purity or the maintenance of pericentromeric region (**S6E Fig**), is the main target. Indeed, one unique advantage of our restriction enzyme combination is that it can also be applied to study pericentromeric regions (**Fig 3B**).

It is important to emphasize that NGS is only one of the many possible downstream applications of the CenRICH method. Here we show that our method to enrich and purify human centromeres is indeed suitable for direct visualization of single DNA molecules. This includes the analysis of replicating DNA fibers aimed at studying replication fork dynamics using techniques as DNA combing (**Fig 5**). Such approaches rely on the usage of DNA probes to label specific regions, like the centromeres, and despite being feasible [42, 54, 55], pose several technical issues. By having a sample with more than half of the DNA fibers of centromeric origin (**Fig 5B**), it is possible to bypass the usage of specific labeling or, depending on the enzyme combination, perform replication studies on specific centromeres. A 25-fold increase in centromeric fibers is particularly relevant for DNA replication studies, given that only a fraction of the fibers will be actively replicating at any given time window. An enrichment in centromeric fibers is even more important in techniques in which the usage of fluorescent probes is not feasible as EM or atomic force microscopy. For example, following *in vivo* psoralen crosslinking, the enriched centromeric DNA can be further processed for EM to study the replication and recombination intermediates at centromeres, as previously done for telomeres [17, 18]. This has the potential to shed light on the architecture and replication intermediates that are present at centromeric regions and help understand how centromeric DNA binding proteins might modulate their topology and structure.

In conclusion, the CenRICH method represents an invaluable tool for the study of human centromeric repeat arrays, particularly useful to compare the same cell line subjected to different experimental conditions. This new development has great possibilities of application and is particularly timely, as the study of centromeric DNA has just entered a new genomic era thanks to the fine mapping and assembly of their repeats [4, 5, 30, 34]. However, we are just scratching the surface of the molecular characterization of centromeric DNA. Indeed, even if human centromeres have been well-characterized thanks to the efforts of the T2T consortium, these data derive mainly from one single cell line, while it is known that centromeres can vary across individuals and can be drastically altered (e.g. length, epigenetic status, organization) in pathological conditions associated with genome instability, such as cancer. We therefore envision that the study of centromeric DNA will be in high demand in the near future, and our CenRICH method can facilitate centromere studies.

## Supporting information

S1 Fig Distribution of centromere DNA after *in silico* digestion of a reference genome. **Related to Fig 1. A-B.** Distribution of centromeric (red) or non-centromeric (blue) base-pair content of predicted fragments according to fragment length after *in silico* digestion of T2T-CHM13v1.0 genome with enzyme combinations SNE (A) and SEB (B). **C**. Distribution of predicted fragment lengths of centromeric fragments after *in silico* digestion of the reference T2T-CHM13v1.0 genome with the SEB enzyme combination. y-axis represents the percentage of centromeric fragments in each length range.
(TIF)

S2 Fig Centromeric DNA is enriched in the high molecular weight fractions, which are deprived of telomeric DNA. Related to Fig 2. A. qPCR analysis showing enrichment in centromeric DNA in the different sucrose fractions after digestion with SEB or SNE enzyme combination. Ct values were normalized to the signal from a ribosomal-DNA-specific primer pair. Fold enrichment is expressed over the undigested unfractionated genomic DNA sample. Bars show means with standard deviation, n = 3. B. Dot-blot to detect abundance of centromeric DNA (measured by signal intensity with a CENP-B box probe, left membranes) or telomeric DNA (right membranes) in different sucrose gradient fractions (F2 to F4; F5+F6 is a pool of fractions F5 and F6) and in unfractionated undigested genomic DNA (gDNA). A specific probe for the Alu repeat was used as a control (middle membranes). In all membranes increasing amounts of DNA were loaded (50, 100 and 200 ng). The top three membranes were loaded with samples digested with SNE combination enzymes (same as Fig 2B), while the bottom three membranes were loaded with samples digested with SNE + telomere specific endonuclease (T-EN). C. Quantification of the telomeric signal from the dot-blot showed in B; signal is reported as a ratio to gDNA. Bars represent the average of the different amounts of DNA. Error bars represent the standard error of the three DNA quantities. D. Agarose gel electrophoresis performed on genomic DNA digested with the SEB combination (top) and corresponding Southern blot after hybridization of the membrane with an α-satellite probe (bottom). “gDNA” represents the unfractionated sample and F1 to F6 represent different fractions. Efficient size separation is shown by the fractionation in sucrose gradient of a molecular weight marker (Gene Ruler 1 Kb). E. Agarose gel electrophoresis and corresponding Southern blots performed on genomic DNA digested with the SNE and SEB combinations, after hybridization with telomeric probe. “gDNA” represents the unfractionated sample and F1 to F6 represent different fractions. A molecular weight marker was used as a control and tested by agarose gel electrophoresis (Gene Ruler 1 Kb) proving the efficiency of sucrose gradient fractionation.
(TIFF)

S3 Fig Southern blots showing enrichment distribution according to molecular weight. **Related to Fig 2. A-E:** Agarose gel electrophoresis and corresponding Southern blot after hybridization with α-satellite probe. F1 to F6 represent the fractions resulting from sucrose gradient fractionation (low to high molecular weight). gDNA represents digested not fractionated DNA. The name of the cell line is reported at the top in each panel. RPE-1 rep2 corresponds to an independent CenRICH experiment aiming at replicating the one of Fig 2D. HCT116 WT and KO represent two genotypes of HCT116 cells, either wild-type or double knock-out for DNMT1 and DNMT3B. RPE-1, DLD-1, CHM13 samples were digested with the SNE enzyme combination; HCT116 samples were digested with the SEB enzyme combination.
(TIF)

S4 Fig Enrichment in centromeric DNA detected by Illumina sequencing. **Related to Fig 3. A**. Quantification of uncut restriction sites identified within Illumina reads after digestion with SNE or SEB enzyme combinations and fractionation (fractions F2, F3, F4). Values are reported as % of the sites identified in the reads from an undigested unfractionated sample (WGS). **B**. Quantification of Illumina reads containing alpha satellite 18-mers, after SNE or SEB digestion and sucrose gradient separation (F2, F3 and F4) and in an undigested sample (WGS). Read counts are reported as a percentage of total reads. **C.** Examples of enrichment profiles in different fractions (F1-2, F3, and F4-6) after SNE digestion and sucrose gradient fractionation of RPE-1 DNA. Enrichment is plotted as log2 ratio over WGS in 2-Kb wide genomic bins. Y-axis ranges between -8 and +8. Genomic coordinates on the T2T-CHM13v1.0 reference are reported on top in Mb. Boundaries of centromeric regions (Cen Region, black bars) and HORs (grey bars) are described in S1 and S3 Tables, respectively. **D**. Enrichment in centromere-derived reads after Illumina sequencing across the different centromeres in fractions F3 and F4 after SEB digestion. Enrichment is expressed as a ratio to the read counts in the WGS sample. **E.** Length of the enrichment domains that overlap with centromeric regions (first column) or HOR arrays (third column). Data refer to RPE-1 DNA that underwent CenRICH with SNE enzyme combination. Second and fourth columns report the length of centromeric region and the cumulative length of HOR arrays on the T2T-CHM13v1.0 reference genome (as defined in S1 and S3 Tables). Lengths are expressed in Mb. The enrichment domains are defined as the regions with an enrichment > 5-fold. The color gradient corresponds to the percentage of the centromeric region or of the HOR array which is covered by the enrichment domain. **F.** Scatter plot and linear regression showing correlation between DLD-1 and RPE-1 in the proportion of HOR arrays that are covered by enrichment domains (fold enrichment > 5). HOR arrays boundaries are defined in S3 Table. Each dot represents one of the 14 centromeres where enrichment is > 5 according to Fig 3C. Dashed lines represent 95% confidence intervals. R^2^ = 0.885, p<0.0001.
(TIF)

S5 Fig Nanopore sequencing and methylation analysis. **Related to Fig 4**. **A.** TapeStation electropherogram profiles of RPE-1 SNE-digested DNA after sucrose gradient fractionation and pooling of fractions F4 to F6, before (red line) and after (blue line) additional size selection by precipitation with the Short Read Eliminator kit. The bulk of DNA is within a peak at ~55 kb. The peak at 100 bp (labelled “lower MW marker” and marked with a grey rectangle) corresponds to a calibrator added for comparison of the two samples. **B.** Distribution of base-pair content of Nanopore reads according to read length. RPE-1 DNA sample after CenRICH with SNE enzyme combination, pool of fractions F4 to F6. **C.** Wenn diagram showing the centromeric CpG sites with an assigned methylation frequency value (ranging from 0 to 100%) in a whole genome Nanopore sequencing (WGS) of RPE-1 cells or in RPE-1 following the CenRICH (F4-6, same as B). **D.** Average methylation frequency across centromeric CpGs in WGS or CenRICH samples (F4-6) from RPE-1 cells. Only sites called in both samples are included. **E.** Scatter plot and linear regression showing correlation in DNA methylation frequencies between WGS and CenRICH(F4-6; same samples as C, D). Only CpGs covered in both samples by at least 10 reads are included. n = 1818 sites. p-value < 0.001. R^2^ = 0.779. **F.** Wenn diagram showing the centromeric CpG sites with an assigned methylation frequency value (ranging from 0 to 100%) in two CenRICH samples from a wild-type (WT) and a DNMT1/3B knock-out (KO) HTC116 cell line. **G.** Distribution of CpG sites based on the minimum coverage. The minimum coverage is expressed as number of Nanopore reads covering the site. Same samples as F. **H.** Scatter plot and linear regression showing correlation in methylation frequencies between WT and KO HTC116 samples (same as F, G, H) after CenRICH. Only CpGs covered in both samples by at least 10 reads are included. n = 67624 sites. p-value < 0.0001. R^2^ = 0.3418. **I.** COBRA analysis comparing methylation level between WT and KO HTC116 cells using primers specific for HSATII repeats, alpha satellite (α-sat), LINE-1 repeats (LINE1) or MAEL gene promoter. The samples without addition of restriction enzyme are shown as control. **J.** Quantification of I. Bars represent the ratio of the intensities between lower (methylated, digested) and higher (unmethylated, undigested) bands. Values are normalized setting WT as 100%. Error bars represent the standard deviation between two replicates.
(TIF)

S6 Fig From DNA combing and replication analysis to an *in silico* digestion of a reference genome with an additional enzyme combination. **Related to Fig 5 and discussion. A.** Graph shows the size distribution of DNA fibers positive or negative to a α-satellite probe, after CenRICH on RPE-1 cells using the SNE combination. Each dot is a DNA fiber. Fibers of less than 10 kb are not analyzed. n = 144 for both conditions. **B.** Example image of a DNA combing on an RPE-1 CenRICH sample (SNE enzyme combination), with some molecules showing CldU incorporation. Most of the DNA fragments are centromeric (labelled in green by a centromeric probe). Scale bar 10 μm. **C.** Size distribution of centromere fibers length as measured by DNA combing in a CenRICH RPE-1 sample digested with SNE combination. NT: untreated. APH: treated with aphidicolin. n = 297 for NT, n = 298 for APH. **D.** Distribution of centromeric (red) or non-centromeric (blue) base-pair content of predicted fragments according to fragment length after *in silico* digestion of T2T-CHM13v1.0 genome with MscI-AseI enzyme combination. **E.** Distribution of predicted centromeric and HSat fragment length after *in silico* digestion of the reference T2T-CHM13v1.0 genome with the MscI-AseI combination (black). y-axis represents the percentage of centromeric fragments in each length range.
(TIF)

S1 Table Genomic coordinates on the T2T-CHM13v1.0 reference genome that define the boundaries of the centromeric regions. (PDF)

S2 Table Genomic coordinates on the T2T-CHM13v1.0 reference genome that define the boundaries of the HSat repeats. (XLSX)

S3 Table Genomic coordinates on the T2T-CHM13v1.0 reference genome that define the boundaries of the HOR arrays. (XLSX)

S4 Table Percentage of centromeric DNA covered by WGS and CenRICH. The table reports the percentage of all HORs sequences included in CenRICH or WGS Illumina sequencing according to the minimum coverage. All HORs reported in S3 Table were divided in 2 Kb bins. The percentage is calculated as the proportion of the bins that have an average coverage of at least the value reported in column one. All calculations are based on the same starting amount of Illumina reads both for CenRICH and WGS (about 7 Gb). The data corresponds to the same datasets as in Fig 3C RPE-1.
(DOCX)

S5 Table Cost estimation of the CenRICH procedure. The cost per sample is derived from supplier prices for the French market as of June 2022, calculated as the cost for extracting 2–3 mg of DNA and obtaining enough enriched material to perform Nanopore, Illumina and DNA combing experiments. The estimation excludes the cost for labor, instrumentation and cell culturing, as they can be extremely variable. The total price range depends on which of the enzyme combinations (SNE or SEB) is used.
(DOCX)
