# Supplementary figures and images for: Enrichment of centromeric DNA from human cells
Source: PLoS Genet. 2022 Jul 19;18(7):e1010306. doi: 10.1371/journal.pgen.1010306 (PMC9295943; doi:10.1371/journal.pgen.1010306)

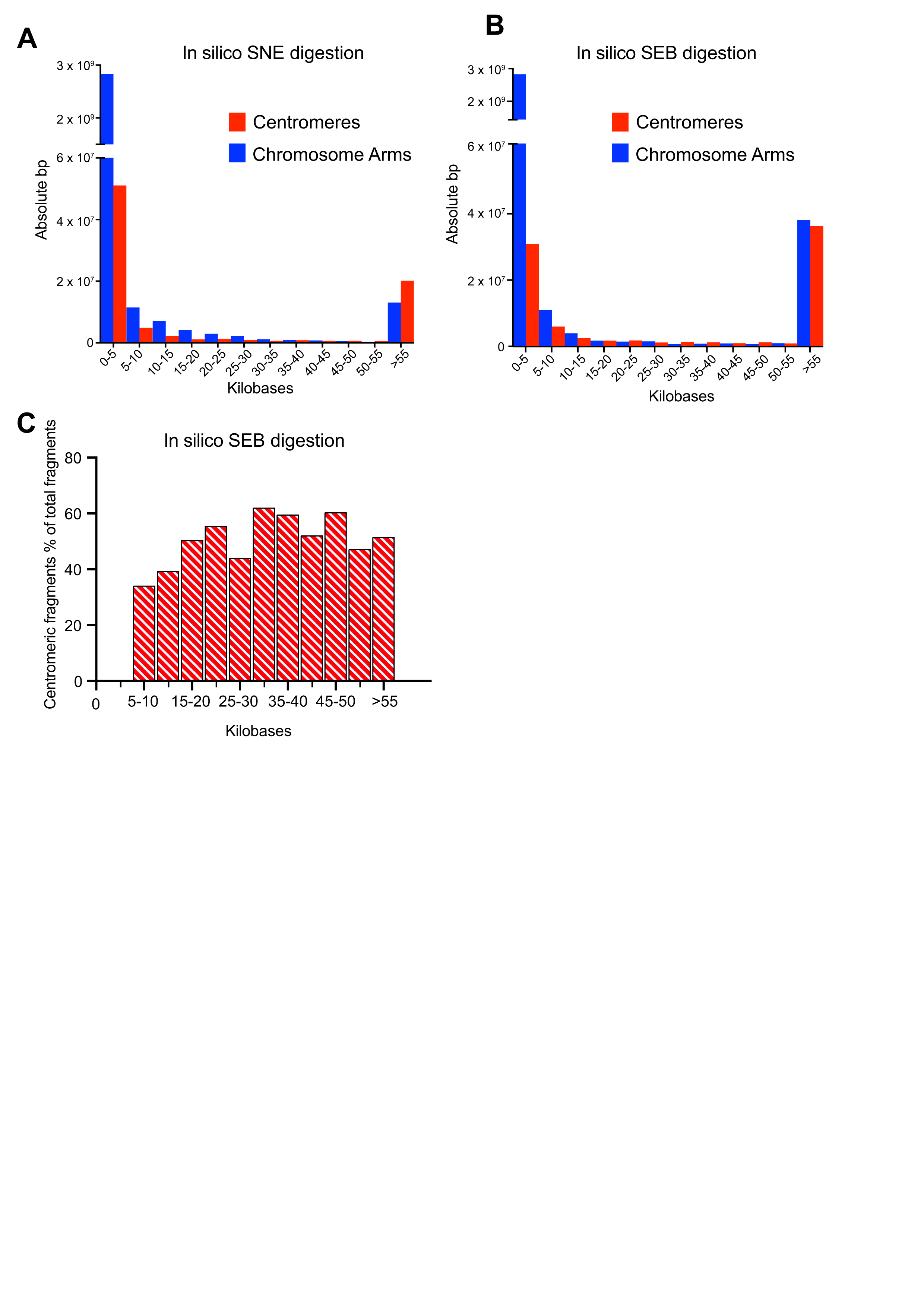

Supplement: S1 Fig — Related to Fig 1. A-B. Distribution of centromeric (red) or non-centromeric (blue) base-pair content of predicted fragments according to fragment length after in silico digestion of T2T-CHM13v1.0 genome with enzyme combinations SNE (A) and SEB (B). C. Distribution of predicted fragment lengths of centromeric fragments after in silico digestion of the reference T2T-CHM13v1.0 genome with the SEB enzyme combination. y-axis represents the percentage of centromeric fragments in each length range. (TIF) [file pgen.1010306.s001.tif]

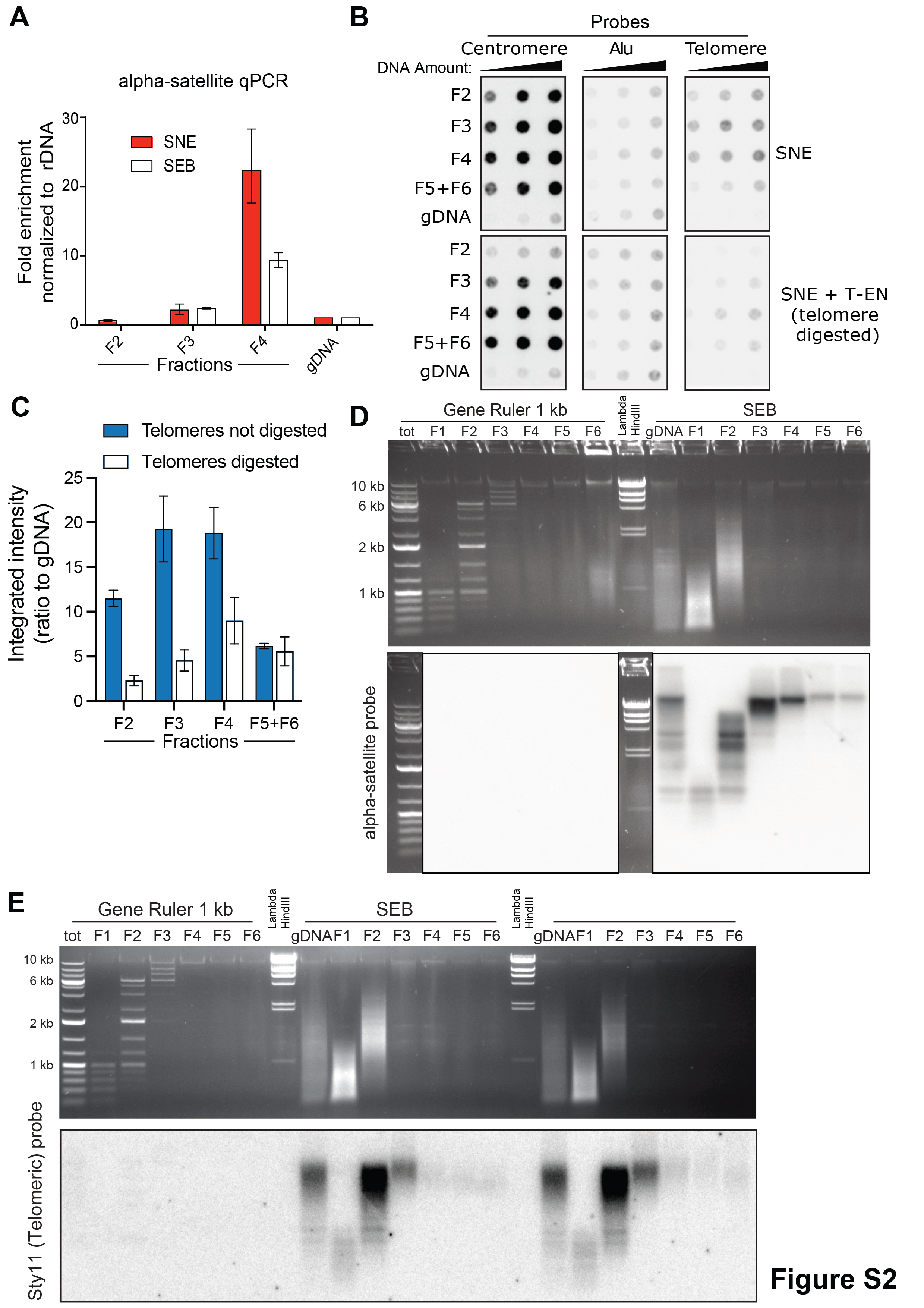

Supplement: S2 Fig — A. qPCR analysis showing enrichment in centromeric DNA in the different sucrose fractions after digestion with SEB or SNE enzyme combination. Ct values were normalized to the signal from a ribosomal-DNA-specific primer pair. Fold enrichment is expressed over the undigested unfractionated genomic DNA sample. Bars show means with standard deviation, n = 3. B. Dot-blot to detect abundance of centromeric DNA (measured by signal intensity with a CENP-B box probe, left membranes) or telomeric DNA (right membranes) in different sucrose gradient fractions (F2 to F4; F5+F6 is a pool of fractions F5 and F6) and in unfractionated undigested genomic DNA (gDNA). A specific probe for the Alu repeat was used as a control (middle membranes). In all membranes increasing amounts of DNA were loaded (50, 100 and 200 ng). The top three membranes were loaded with samples digested with SNE combination enzymes (same as Fig 2B), while the bottom three membranes were loaded with samples digested with SNE + telomere specific endonuclease (T-EN). C. Quantification of the telomeric signal from the dot-blot showed in B; signal is reported as a ratio to gDNA. Bars represent the average of the different amounts of DNA. Error bars represent the standard error of the three DNA quantities. D. Agarose gel electrophoresis performed on genomic DNA digested with the SEB combination (top) and corresponding Southern blot after hybridization of the membrane with an α-satellite probe (bottom). “gDNA” represents the unfractionated sample and F1 to F6 represent different fractions. Efficient size separation is shown by the fractionation in sucrose gradient of a molecular weight marker (Gene Ruler 1 Kb). E. Agarose gel electrophoresis and corresponding Southern blots performed on genomic DNA digested with the SNE and SEB combinations, after hybridization with telomeric probe. “gDNA” represents the unfractionated sample and F1 to F6 represent different fractions. A molecular weight marker was used as a control a [file pgen.1010306.s002.tiff]

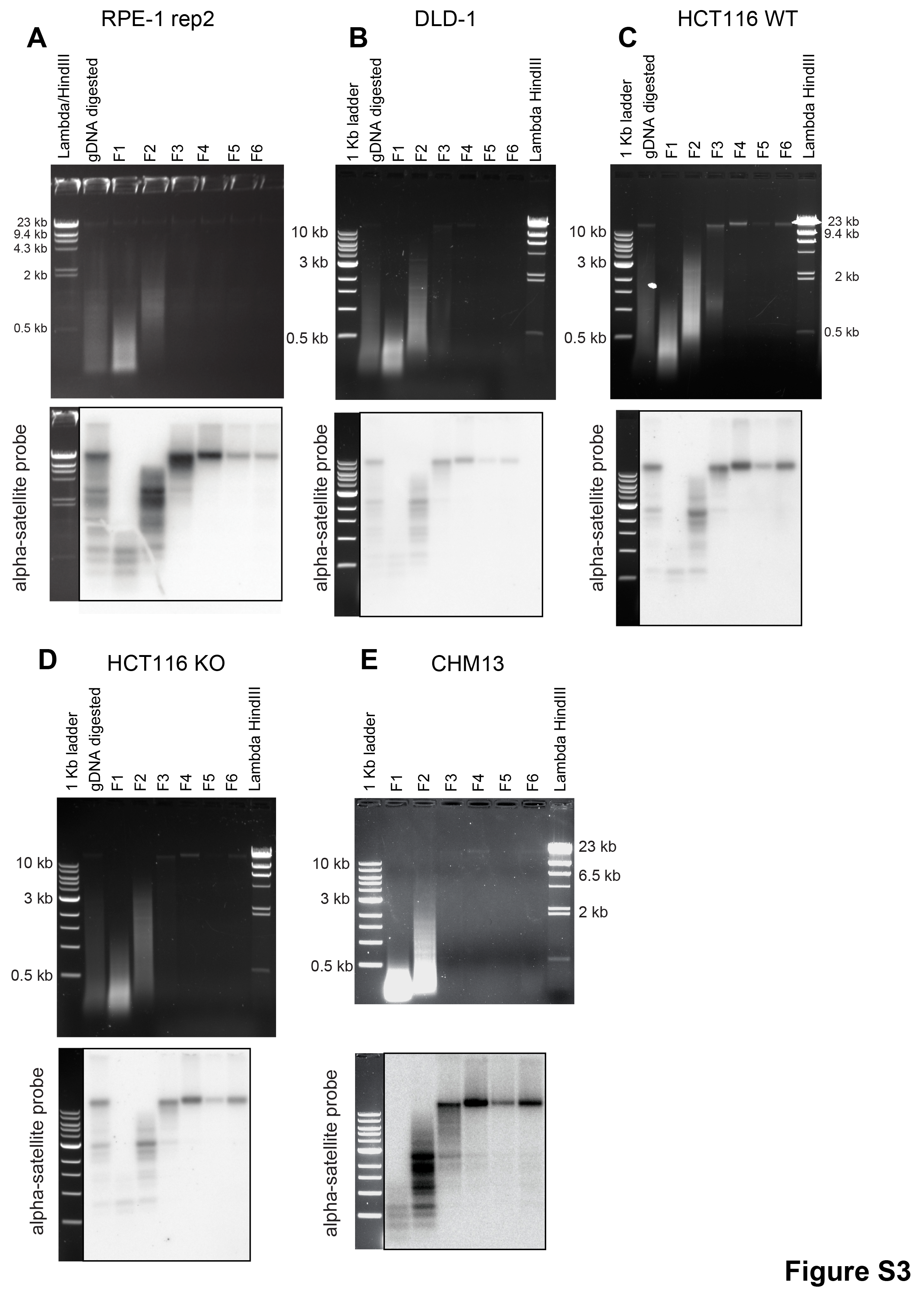

Supplement: S3 Fig — Related to Fig 2. A-E: Agarose gel electrophoresis and corresponding Southern blot after hybridization with α-satellite probe. F1 to F6 represent the fractions resulting from sucrose gradient fractionation (low to high molecular weight). gDNA represents digested not fractionated DNA. The name of the cell line is reported at the top in each panel. RPE-1 rep2 corresponds to an independent CenRICH experiment aiming at replicating the one of Fig 2D. HCT116 WT and KO represent two genotypes of HCT116 cells, either wild-type or double knock-out for DNMT1 and DNMT3B. RPE-1, DLD-1, CHM13 samples were digested with the SNE enzyme combination; HCT116 samples were digested with the SEB enzyme combination. (TIF) [file pgen.1010306.s003.tif]

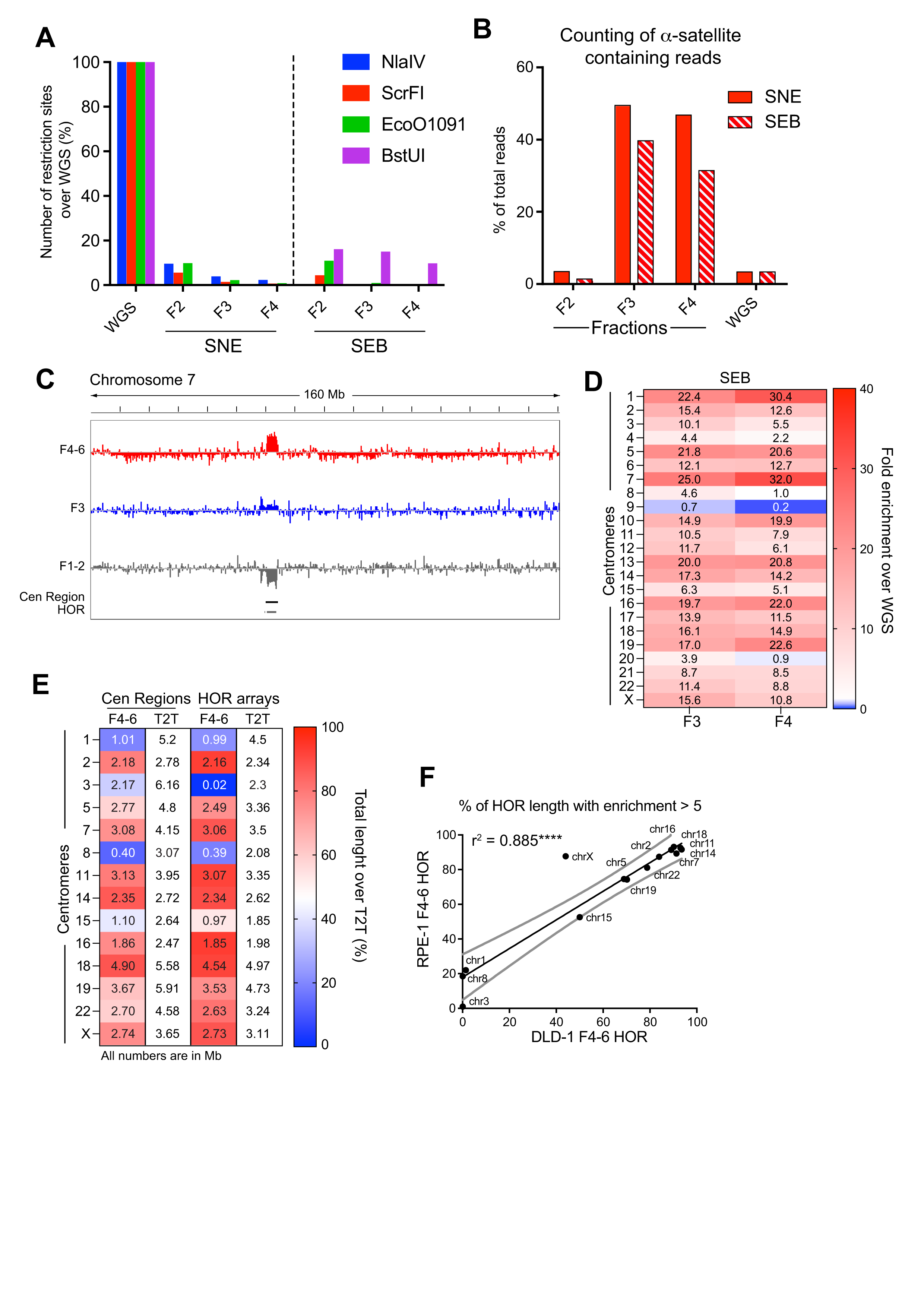

Supplement: S4 Fig — Related to Fig 3. A. Quantification of uncut restriction sites identified within Illumina reads after digestion with SNE or SEB enzyme combinations and fractionation (fractions F2, F3, F4). Values are reported as % of the sites identified in the reads from an undigested unfractionated sample (WGS). B. Quantification of Illumina reads containing alpha satellite 18-mers, after SNE or SEB digestion and sucrose gradient separation (F2, F3 and F4) and in an undigested sample (WGS). Read counts are reported as a percentage of total reads. C. Examples of enrichment profiles in different fractions (F1-2, F3, and F4-6) after SNE digestion and sucrose gradient fractionation of RPE-1 DNA. Enrichment is plotted as log2 ratio over WGS in 2-Kb wide genomic bins. Y-axis ranges between -8 and +8. Genomic coordinates on the T2T-CHM13v1.0 reference are reported on top in Mb. Boundaries of centromeric regions (Cen Region, black bars) and HORs (grey bars) are described in S1 and S3 Tables, respectively. D. Enrichment in centromere-derived reads after Illumina sequencing across the different centromeres in fractions F3 and F4 after SEB digestion. Enrichment is expressed as a ratio to the read counts in the WGS sample. E. Length of the enrichment domains that overlap with centromeric regions (first column) or HOR arrays (third column). Data refer to RPE-1 DNA that underwent CenRICH with SNE enzyme combination. Second and fourth columns report the length of centromeric region and the cumulative length of HOR arrays on the T2T-CHM13v1.0 reference genome (as defined in S1 and S3 Tables). Lengths are expressed in Mb. The enrichment domains are defined as the regions with an enrichment > 5-fold. The color gradient corresponds to the percentage of the centromeric region or of the HOR array which is covered by the enrichment domain. F. Scatter plot and linear regression showing correlation between DLD-1 and RPE-1 in the proportion of HOR arrays that are covered by enrichment domains (fold enric [file pgen.1010306.s004.tif]

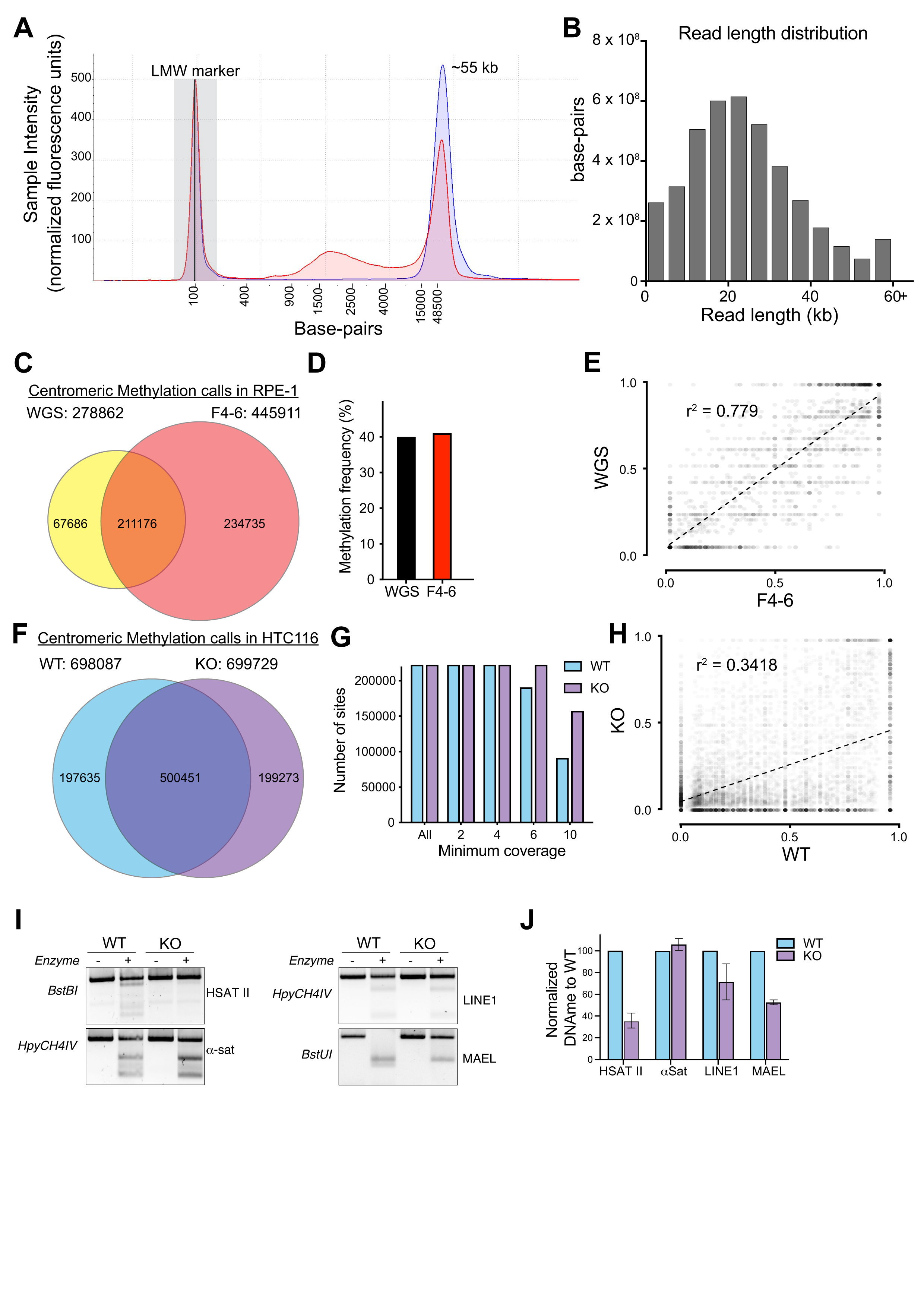

Supplement: S5 Fig — Related to Fig 4. A. TapeStation electropherogram profiles of RPE-1 SNE-digested DNA after sucrose gradient fractionation and pooling of fractions F4 to F6, before (red line) and after (blue line) additional size selection by precipitation with the Short Read Eliminator kit. The bulk of DNA is within a peak at ~55 kb. The peak at 100 bp (labelled “lower MW marker” and marked with a grey rectangle) corresponds to a calibrator added for comparison of the two samples. B. Distribution of base-pair content of Nanopore reads according to read length. RPE-1 DNA sample after CenRICH with SNE enzyme combination, pool of fractions F4 to F6. C. Wenn diagram showing the centromeric CpG sites with an assigned methylation frequency value (ranging from 0 to 100%) in a whole genome Nanopore sequencing (WGS) of RPE-1 cells or in RPE-1 following the CenRICH (F4-6, same as B). D. Average methylation frequency across centromeric CpGs in WGS or CenRICH samples (F4-6) from RPE-1 cells. Only sites called in both samples are included. E. Scatter plot and linear regression showing correlation in DNA methylation frequencies between WGS and CenRICH(F4-6; same samples as C, D). Only CpGs covered in both samples by at least 10 reads are included. n = 1818 sites. p-value < 0.001. R2 = 0.779. F. Wenn diagram showing the centromeric CpG sites with an assigned methylation frequency value (ranging from 0 to 100%) in two CenRICH samples from a wild-type (WT) and a DNMT1/3B knock-out (KO) HTC116 cell line. G. Distribution of CpG sites based on the minimum coverage. The minimum coverage is expressed as number of Nanopore reads covering the site. Same samples as F. H. Scatter plot and linear regression showing correlation in methylation frequencies between WT and KO HTC116 samples (same as F, G, H) after CenRICH. Only CpGs covered in both samples by at least 10 reads are included. n = 67624 sites. p-value < 0.0001. R2 = 0.3418. I. COBRA analysis comparing methylation level between WT and KO HTC116 cells [file pgen.1010306.s005.tif]

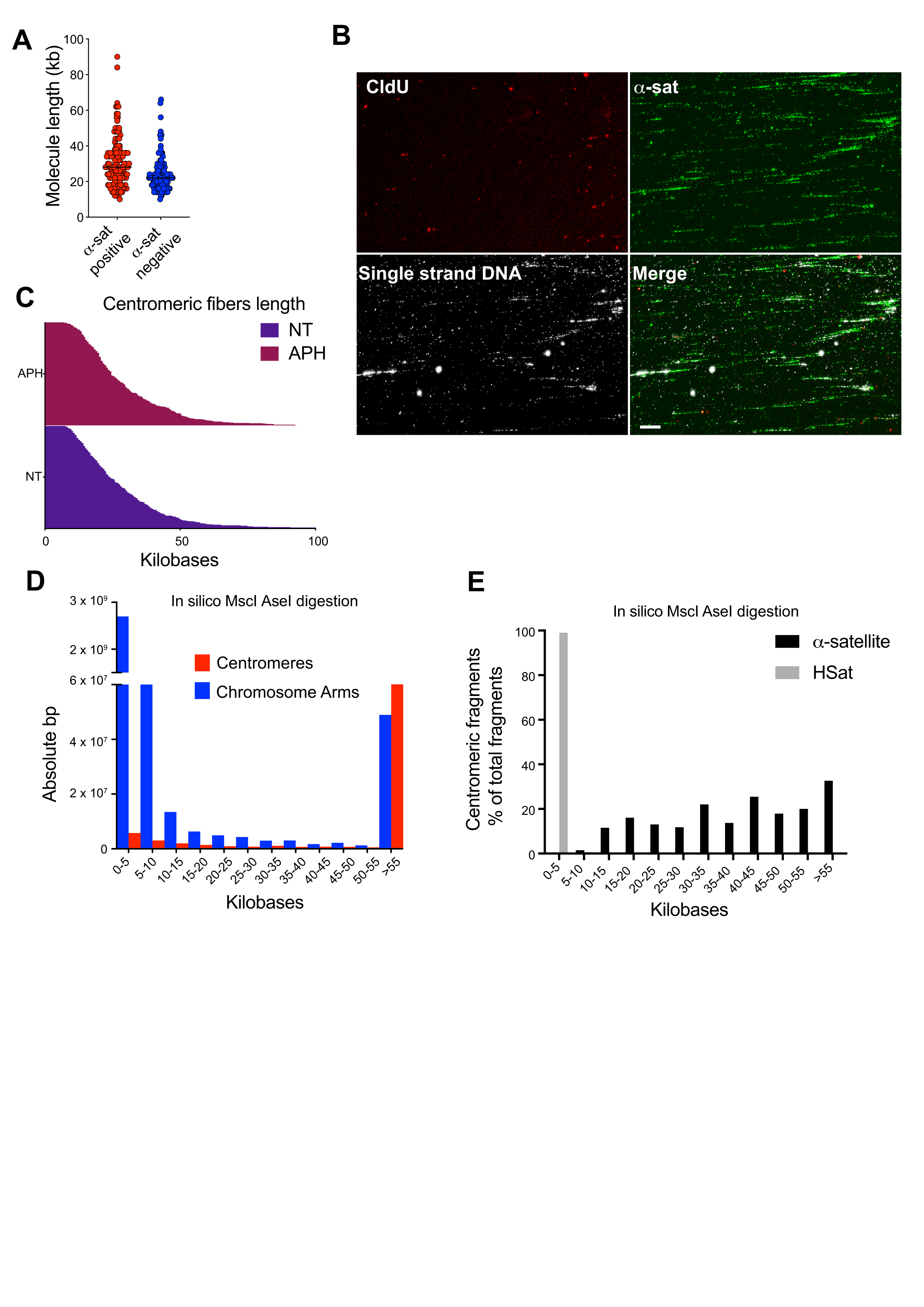

Supplement: S6 Fig — Related to Fig 5 and discussion. A. Graph shows the size distribution of DNA fibers positive or negative to a α-satellite probe, after CenRICH on RPE-1 cells using the SNE combination. Each dot is a DNA fiber. Fibers of less than 10 kb are not analyzed. n = 144 for both conditions. B. Example image of a DNA combing on an RPE-1 CenRICH sample (SNE enzyme combination), with some molecules showing CldU incorporation. Most of the DNA fragments are centromeric (labelled in green by a centromeric probe). Scale bar 10 μm. C. Size distribution of centromere fibers length as measured by DNA combing in a CenRICH RPE-1 sample digested with SNE combination. NT: untreated. APH: treated with aphidicolin. n = 297 for NT, n = 298 for APH. D. Distribution of centromeric (red) or non-centromeric (blue) base-pair content of predicted fragments according to fragment length after in silico digestion of T2T-CHM13v1.0 genome with MscI-AseI enzyme combination. E. Distribution of predicted centromeric and HSat fragment length after in silico digestion of the reference T2T-CHM13v1.0 genome with the MscI-AseI combination (black). y-axis represents the percentage of centromeric fragments in each length range. (TIF) [file pgen.1010306.s006.tif]
