# Supplementary material for: Enrichment of centromeric DNA from human cells
Source: PLoS Genet. 2022 Jul 19;18(7):e1010306. doi: 10.1371/journal.pgen.1010306 (PMC9295943; doi:10.1371/journal.pgen.1010306)
Supplement: S1 Table — (PDF) [file pgen.1010306.s007.pdf]

**Table S1:** Genomic coordinates on the T2T-CHM13v1.0 reference genome that define the boundaries of the centromeric regions.

| Chromosome | start | end |
|---|---|---|
| chr1 | 121619339 | 126824297 |
| chr2 | 92297388 | 95077534 |
| chr3 | 90339864 | 96498631 |
| chr4 | 49705248 | 55303286 |
| chr5 | 46161723 | 50962189 |
| chr6 | 58243527 | 61413737 |
| chr7 | 60041897 | 64188432 |
| chr8 | 43846453 | 46920432 |
| chr9 | 44938599 | 47609552 |
| chr10 | 39068394 | 42061834 |
| chr11 | 50537301 | 54487964 |
| chr12 | 34485615 | 37369869 |
| chr13 | 16220369 | 18249463 |
| chr14 | 10125077 | 12841225 |
| chr15 | 15683284 | 18327600 |
| chr16 | 35743659 | 38213682 |
| chr17 | 23407665 | 27656858 |
| chr18 | 15542843 | 21125452 |
| chr19 | 24352654 | 30261056 |
| chr20 | 26281854 | 29953422 |
| chr21 | 11699867 | 12078398 |
| chr22 | 12326835 | 16903875 |
| chrX | 57593566 | 61247455 |
