# Supplementary material for: Enrichment of centromeric DNA from human cells
Source: PLoS Genet. 2022 Jul 19;18(7):e1010306. doi: 10.1371/journal.pgen.1010306 (PMC9295943; doi:10.1371/journal.pgen.1010306)
Supplement: S4 Table — The table reports the percentage of all HORs sequences included in CenRICH or WGS Illumina sequencing according to the minimum coverage. All HORs reported in S3 Table were divided in 2 Kb bins. The percentage is calculated as the proportion of the bins that have an average coverage of at least the value reported in column one. All calculations are based on the same starting amount of Illumina reads both for CenRICH and WGS (about 7 Gb). The data corresponds to the same datasets as in Fig 3C RPE-1. (DOCX) [file pgen.1010306.s010.docx]

| Minimum coverage | CenRICH | WGS |
| --- | --- | --- |
| 1X | 79.8 % | 52.3 % |
| 2X | 65.9 % | 14.6 % |
| 3X | 58.8 % | 4.6 % |
| 5X | 51.4 % | 1.0 % |
| 10X | 44.2 % | 0.2 % |
| 20X | 30.4 % | 0 |
| 30X | 17.3 % | 0 |
| 50X | 4.3 % | 0 |

**S4 Table:** **Percentage of centromeric DNA covered by WGS and CenRICH.** The table reports the percentage of all HORs sequence included in CenRICH or WGS Illumina sequencing according to the minimum coverage. All HORs reported in Table S3 were divided in 2 Kb bins. The percentage is calculated as the proportion of the bins that have an average coverage of at least the value reported in column one. All calculations are based on the same starting amount of Illumina reads both for CenRICH and WGS (about 7 Gb). The data corresponds to the same datasets as in Figure 3C RPE-1.
