# Supplementary material for: Enrichment of centromeric DNA from human cells
Source: PLoS Genet. 2022 Jul 19;18(7):e1010306. doi: 10.1371/journal.pgen.1010306 (PMC9295943; doi:10.1371/journal.pgen.1010306)
Supplement: S5 Table — The cost per sample is derived from supplier prices for the French market as of June 2022, calculated as the cost for extracting 2–3 mg of DNA and obtaining enough enriched material to perform Nanopore, Illumina and DNA combing experiments. The estimation excludes the cost for labor, instrumentation and cell culturing, as they can be extremely variable. The total price range depends on which of the enzyme combinations (SNE or SEB) is used. (DOCX) [file pgen.1010306.s011.docx]

| Item | Cost per sample (€) | Supplier | Cat # |
| --- | --- | --- | --- |
| Tris Base | 0.006 | Euromedex | 200923-A |
| Sodium Chloride | 0.011 | Fisher Scientific | 10092740 |
| EDTA | 0.038 | Thermo Scientific | 15575020 |
| SDS | 0.138 | Euromedex | EU0660 |
| Proteinase K | 9.96 | Thermo Scientific | 25530049 |
| RNase A | 1.88 | Thermo Scientific | 12091021 |
| Phenol Chloroform Isoamyl alcohol | 48.60 | Sigma Aldrich | 77617 |
| Chloroform | 4.80 | Fisher Scientific | 15643700 |
| Sodium Acetate | 0.154 | Fisher Scientific | 11337267 |
| Isopropanol | 3.584 | Fisher Scientific | 15653990 |
| Ethanol | 0.46 | VWR | 20821 |
| Total DNA extraction | **69.63** |  |  |
|  |  |  |  |
| NlaIV | 117.3 | NEB | R0126S |
| EcoO109I | 11 | NEB | R0503S |
| BstUI | 21.42 | NEB | R0518S |
| ScrFI | 23.2 | NEB | R0110S |
| Total DNA digestion: |  |  |  |
| SNE | 151.5 |  |  |
| SEB | 55.62 |  |  |
|  |  |  |  |
| Sucrose | 0.74 | Fisher Scientific | 10638403 |
| Ultra-Clear centrifuge tubes | 22.31 | Beckman Coulter | 344058 |
| Amicon Ultra-2 Centrifugal Filter | 34.91 | Merck Milipore | UFC810024 |
| Amicon Ultra-0.5 Centrifugal Filter | 19.13 | Merck Milipore | UFC500396 |
| SRE kit | 18.75 | Circulomics | SS-100-101-01 |
| Total size fractionation | 95.84 |  |  |
|  |  |  |  |
| Total price | 221 - 317 |  |  |

**S5 Table**: **Estimation of the cost of the CenRICH procedure**. The cost per sample is derived from supplier prices for the French market as of June 2022, calculated as the cost for extracting 2-3 mg of DNA and obtaining enough enriched material to perform Nanopore, Illumina and DNA combing experiments. The estimation excludes the cost for labor, instrumentation and cell culturing, as they can be extremely variable. The total price range depends on which of the enzyme combinations (SNE or SEB) is used.
